# Two chronically misdiagnosed patients infected with *Nocardia cyriacigeorgica* accurately diagnosed by whole genome resequencing

**DOI:** 10.3389/fcimb.2022.1032669

**Published:** 2022-10-12

**Authors:** Anqing Liu, Xiaojin Liu, Yunwei Lu, Zhan Gao, Ruixiang Tang, Yang Huang, Liheng Zheng, Zhenxin Fan, Miao He

**Affiliations:** ^1^ Institute of Blood Transfusion, Chinese Academy of Medical Sciences & Peking Union Medical College, Chengdu, China; ^2^ Sichuan Blood Safety and Blood Substitute International Science and Technology Cooperation Base, Chengdu, China; ^3^ Hebei Provincial Key Laboratory of Lung Disease, Hebei Chest Hospital, Shijiazhuang, China; ^4^ Key Laboratory of Bioresources and Eco−Environment (Ministry of Education), College of Life Sciences, Sichuan University, Chengdu, China

**Keywords:** *nocardia cyriacigeorgica*, bacterial morphology, genome-wide re-sequencing, phylogenomics, infection

## Abstract

Nocardiosis is a rare but life-threatening infection particularly affecting immuno-compromised hosts, causing localized or systemic suppurative disease usually in human beings. *Nocardia* species, as the pathogen of nocardiosis, are difficult to differentiate because of their complex colony morphological features. In this study, we describe two patients who had been misdiagnosed for a long time infected with *Nocardia cyriacigeorgica* with completely different morphology were accurately diagnosed. Single colonies were analyzed by Gram staining, acid-fast stain, mass spectrometry and whole genome resequencing (WGRS). These two bacterial, strains L5.53 and L5.54, were found to be Gram-negative and acid-fast-weak positive. Blood sample culturing of strain L5.53 yielded white colonies, which were like a layer of hoarfrost, while colonies of L5.54 were yellow, rough, slightly convex. The two strains were identified as *Nocardia* sp. by mass spectrometry, and WGRS accurately determined them as *N. cyriacigeorgica.* After medical treatment, one patient was cured and the other was still receiving treatment in the hospital. It can be seen that *Nocardia* sp. cannot be accurately classified and identified only by phenotypic tests such as bacterial morphological differences, so it is necessary to identify *Nocardia* spp. with phenotypic tests in combination with other molecular biology technologies, such as WGRS.

## Introduction

*Nocardia* species are strictly aerobic bacteria with positive or variable gram staining and weakly positive acid-fast staining, belonging to phylum Actinobacteria, class Actinobacteria, order Actinomycetales, and family Nocardiaceae. They widely exist in soil and in wet biotopes and are considered as opportunistic pathogens (Sorrell et al., 2010). At the time of writing, the genus contains 121 species with validly published names (http://www.bacterio.net/nocardia.html), typically including the common pathogens such as *N. asteroides*, *N. farcinica*, *N. nova*, *N. transvalensis*, *N. brasiliensis*, *N. abscessus*, and *N. cyricigeorgica* (Brown-Elliott et al., 2006; Uhde et al., 2010). Until now, the diagnosis of nocardiosis still relies on the isolation and identification of microorganisms from the site of infection (Weng et al., 2020). However, microbiological detection of *Nocardia* spp. is time-consuming, due to their slow growth, it usually takes several days until they are identified (Du et al., 2016) and experienced microbiologists are required. Because of the complex colony morphological features of *Nocardia* spp., including ranging from smooth to rough with clear margined to irregular colonies, various colors, the presence of fragmenting hyphal forms and characteristic presence of short chain spores (Dhakal et al., 2019), it is difficult to differentiate them by bacterial morphological examination. What's more, only limited species in genus *Nocardia* can be distinguished by microbiological detection, while closely related species may exhibit different epidemiology, pathogenicity, and susceptibility to antibiotics (Almeida and Araujo, 2013). Therefore, there is an urgent need for new methods to accurately classify and identify *Nocardia* spp., and to reveal the differences in biological characteristics between different species, so as to facilitate precise treatments. In the present study, we confirmed the two strains isolated from two long-term misdiagnosed patients were the same one by WGRS, which had different morphology, and explored the reasons for the bacterial morphological differences, their pathogenicity, drug resistance, and virulence factors at the genetic level.

## Case description

**Figure 1:**
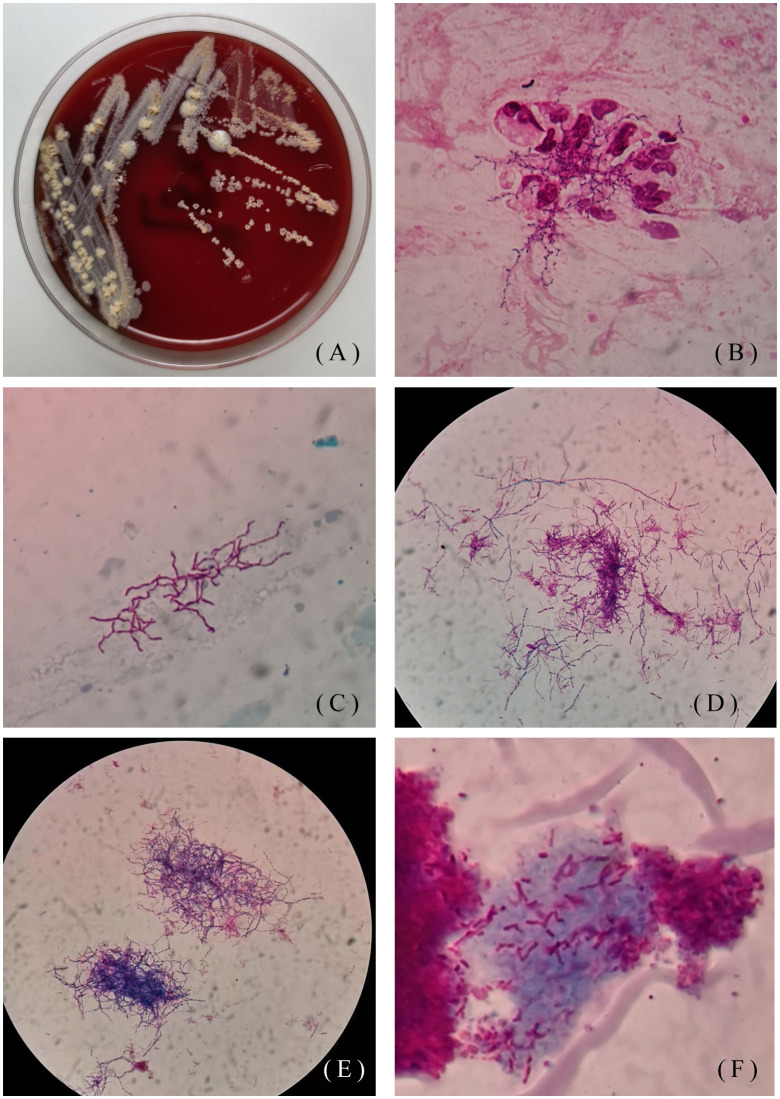
Colony morphology of isolate L5.53. **(A)** Colony morphology of L5.53 on blood agar plates. The colonies were yellow, raised, rough and opaque on blood agar plates; **(B)** Gram staining of strain L5.53. The sputum samples of strain L5.53 were directly stained with gram. The bacteria were curved and beaded with right-angled branches visible; **(C)** Acid-fast staining of sputum specimens of isolate L5.53. Acid-fast staining was weakly positive; **(D)** Acid-fast staining of strain L5.53 after blood plate cultured. Bacterial acid-fast staining was weakly positive, the bacteria became different in length, and the right-angled branches were not obvious; **(E)** Gram staining of strain L5.53 cultured on blood plates. The bacteria became different in length and branching was not obvious; **(F)** Strain L5.53 cultured in 960 tuberculosis liquid medium. The strain L5.53 on the blood plate was cultured in 960 tuberculosis liquid medium, and the bacteria were cut short and indistinguishable from tuberculosis.

One patient was a 48-year-old man. He went to the Hebei Chest Hospital for the first time in 2018, with a history of cough and sputum (yellow sticky sputum) for more than 7 years, which worsened for half a month. He suffered from insomnia for more than 10 years and took diazepam and estazolam orally all year round. On examination, the patient had low breath sounds in both lungs, and dry and moist rales could be heard. Sputum samples were negative for acid-fast staining and positive for weak acid-fast staining. Tuberculin test (PPD) was negative. *Klebsiella pneumoniae* and *Candida albicans* grew on a sputum specimen. He was diagnosed with chronic obstructive pulmonary disease, diffuse panbronchiolitis, and insomnia. The patient was treated with piperacillin-tazobactam and levofloxacin for 7 days. After taking compound sulfamethoxazole for half a year, the patient was cured. In 2019, the patient was admitted to the hospital due to aggravated cough and expectoration. Seven years after misdiagnosis, *Nocardia* spp. (isolate L5.53) was discovered by sputum culture and mass spectrometry on May 15, 2019. Strain L5.53 was isolated from the sputum samples of the patients, and grew on blood agar plate, Lowenstein-Jenden medium and 960 tuberculosis liquid medium, which were curved under the microscope and not straight(**Figures 1A–F**).

**Figure 2:**
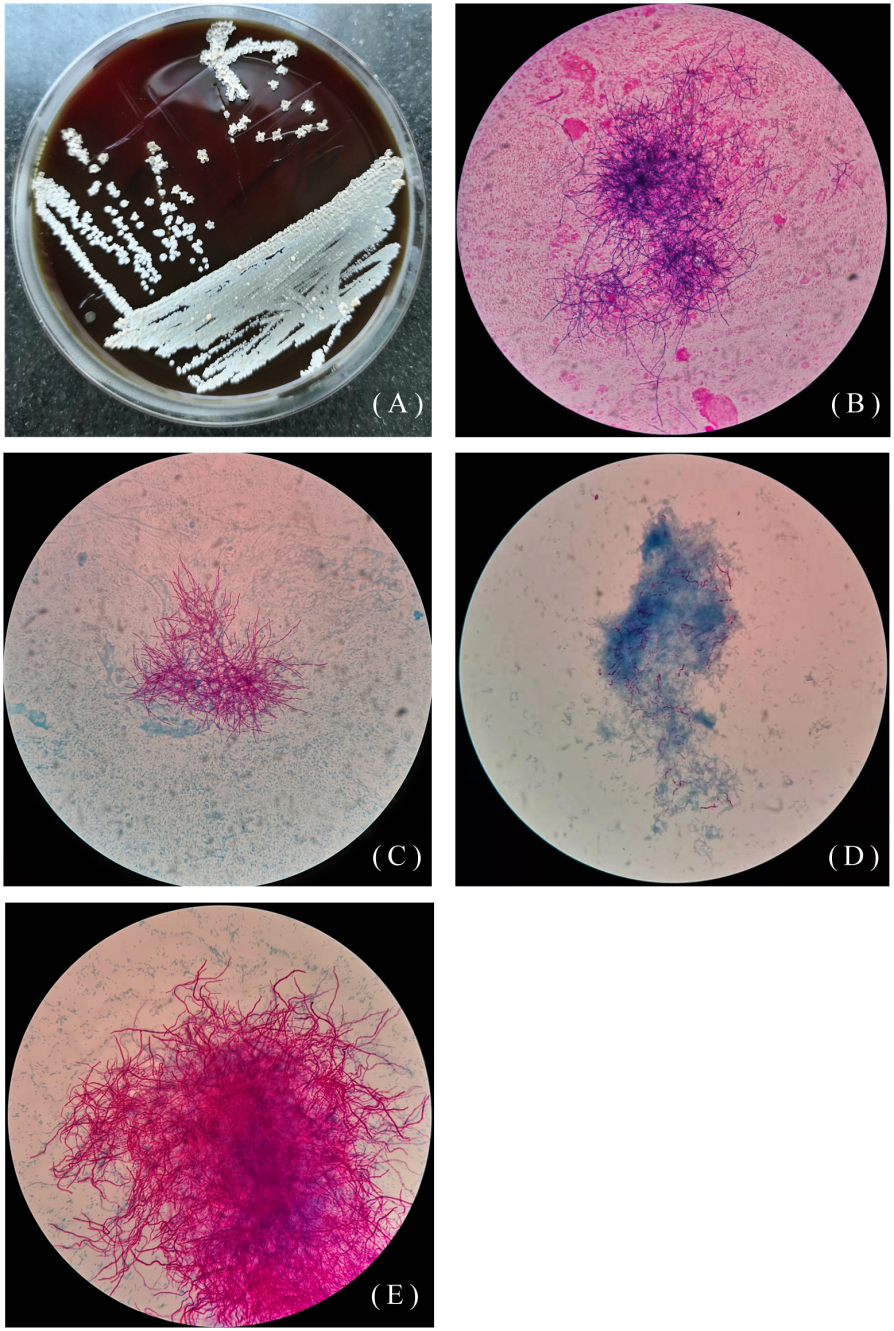
Colony morphology of isolate L5.54. **(A)** Colony morphology of L5.54 on blood agar plates. The colonies were white, rough and opaque, like a layer of hoarfrost on blood agar plates; **(B)** Gram staining of strain L5.54. After Gram staining of sputum specimens, it was observed that bacterial hyphae were stretched; **(C)** Acid-fast staining of sputum specimens of isolate L5.54. Acid-fast staining was weakly positive; **(D)** Acid-fast staining of strain L5.54 after blood plate cultured. Bacterial acid-fast staining was weakly positive, and the bacterial hyphae were very short; **(E)** Strain L5.54 cultured in 960 tuberculosis liquid medium. The strain L5.54 on the blood plate was cultured in 960 tuberculosis liquid medium, and the short bacteria reverted to long filaments.

The other 46-year-old female patient presented with recurrent cough, sputum, and hemoptysis for 19 years, lower extremity pain for 2 months, and was allergic to amoxicillin. In the past 19 years, she had been hospitalized for anti-infective treatment several times, using the drugs including ceftriaxone, levofloxacin, aztreonam, dexamethasone. The sputum specimen was positive for weak acid-fast staining and negative for tuberculosis. A sputum specimen was taken and colonies of *Pseudomonas aeruginosa* and *Nocardia* spp. were found on June 26, 2019. As shown in **Figures 2A–E**, *Nocardia* spp. (isolate L5.54) were stretched and filamentous, cultured on blood agar plate, Lowenstein-Jenden medium and 960 tuberculosis liquid medium. After 13 years of misdiagnosis, the patient was diagnosed with bronchiectasis secondary infection and pulmonary nocardiosis with cavitation. After treatment with piperacillin-tazobactam combined with levofloxacin and compound sulfamethoxazole, she developed compound sulfamethoxazole fever and allergic to piperacillin-tazobactam. On August 1, 2019, the drug was adjusted to levofloxacin 0.5 1/day, clarithromycin 0.25 2/day, ambroxol 30mg 2/day, and compound ipratropium bromide 2.5mg aerosol inhalation 2/day. On October 23, 2019, bronchoscopy showed actinomycetes. The patient was infected with *N. cyriacigeorgica* and *Haemophilus influenzae*, and was given oral therapy with moxifloxacin combined with clarithromycin. On October 25, 2019, levofloxacin combined with clarithromycin was used for treatment, and then on December 10, 2019, it was adjusted to moxifloxacin and aztreonam. Due to various drug allergies, the treatment effect was not good, and she was still under anti-infective treatment.

## Materials and methods

### DNA extraction

Genomic DNA of isolates L5.53 and L5.54 were extracted from pure culture following the protocol adopted by HiPure Fungal DNA Mini Kit. The purity and integrity of the DNA were detected by agarose gel electrophoresis, and quantified by Qubit.

### Library construction

A 10K SMRT Bell library was constructed using the SMRT bell™ Template kit (version 1.0), and the NEBNext®Ultra™ DNA Library Prep Kit for Illumina (NEB, USA) was used to construct a library of 350bp small fragment. The constructed libraries were quantified by Qubit, and the insert size was detected by Agilent 2100.The effective concentration of the expected library was accurately quantified by Q-PCR to ensure the quality of the library.

### Next generation sequencing

After the library check was qualified, different libraries were sequenced by PacBio Sequel and Illumina NovaSeq PE150 according to the effective concentration and target data volume. The quality of the sequence reads of Illumina NovaSeq PE150 was assessed by Q-value (Q20 and Q30).

### Genome assembly and genotype cluster analysis

SMRT Link v5.0.1 software (https://www.pacb.com/support/software-downloads/) was used to assemble the reads. N50 was used to assess the quality of the assembly. Genotype cluster analysis was performed on 117 protein sequences of genus *Nocardia*, including the protein sequences of isolate L5.53 and L5.54, by the software orthofinder, to construct an evolutionary tree. The coding gene sequences of isolate L5.53 and L5.54 were aligned using mummer software. With the LASTAL and JCVI Python, the coding gene sequences of L5.53, L5.54, *N. cyriacigeorgica* GUH-2 as well as *N. farcinica* were compared for collinearity.

### Genome functional analysis

Coding gene predictions were performed on newly sequenced genomes using GeneMarkS (Version 4.17) software. Gene function annotation was performed in GO, KEGG, COG, NCBI-nr, Pfam, TCDB and SwissProt. The pathogen-host interaction database (PHI) was used to annotate L5.53 and L5.54 to find genes related to the pathogenicity of pathogenic bacteria. By using Diamond software, the amino acid sequences of the target species were compared with Antibiotic Resistance Genes Database (ARDB) (Liu and Pop, 2009) and Comprehensive Antibiotic Research Database (CARD) (Alcock et al., 2020) to study the drug resistance genes of isolate L5.53 and L5.54, and compared with the VFDB database (Chen et al., 2005) to analyze virulence factors.

## Results

**Figure 3:**
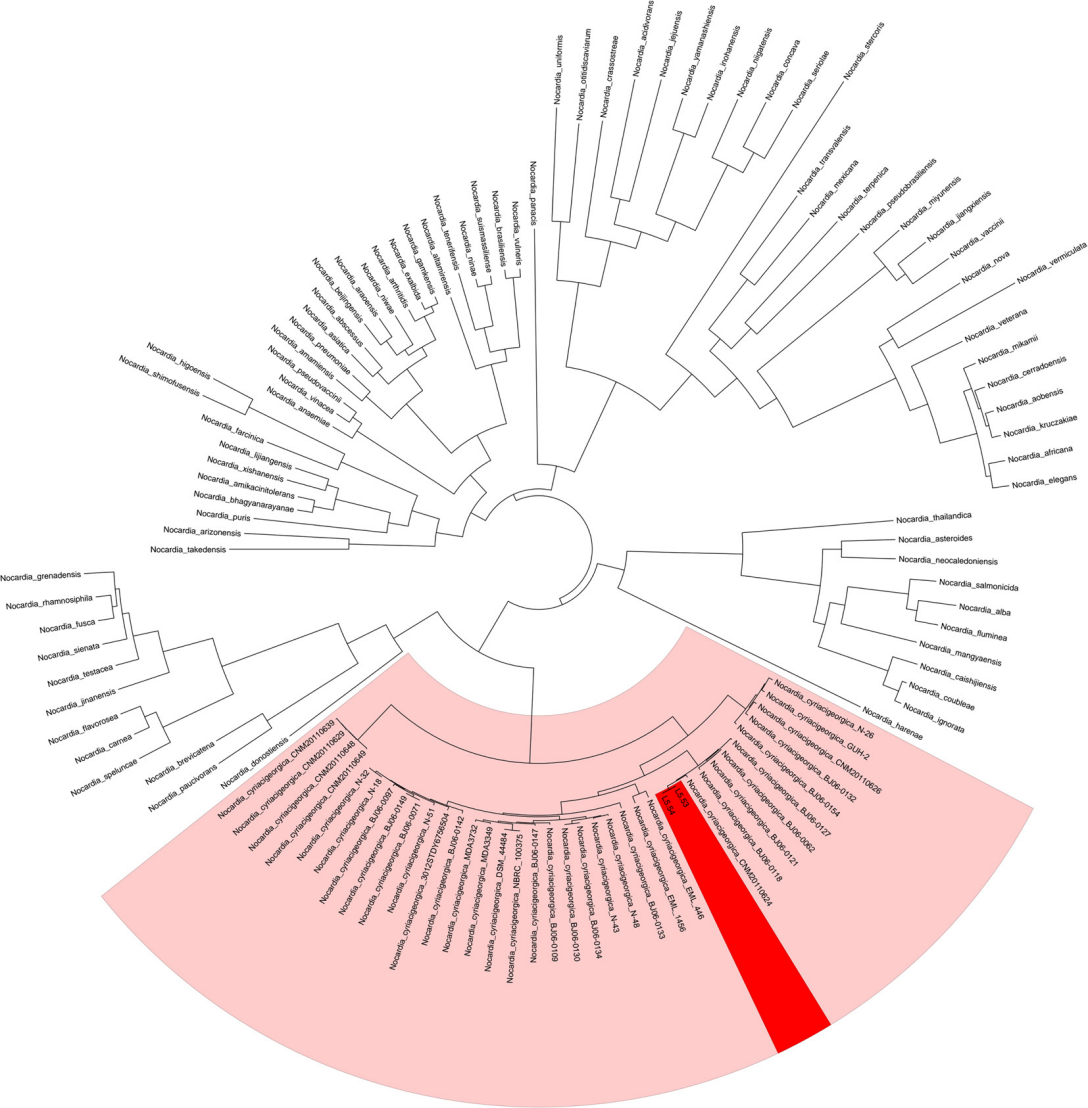
Gene family cluster analysis evolutionary tree of strains L5.53 and L5.54. The 117 protein sequences of genus *Nocardia*, including samples L5.53 and L5.54, were clustered and the evolutionary tree was constructed. It could be seen that L5.53 and L5.54 were *Nocardia cyriacigeorgica*, which formed a robust supported clade within *N. cyriacigeorgica*.

**Figure 4:**
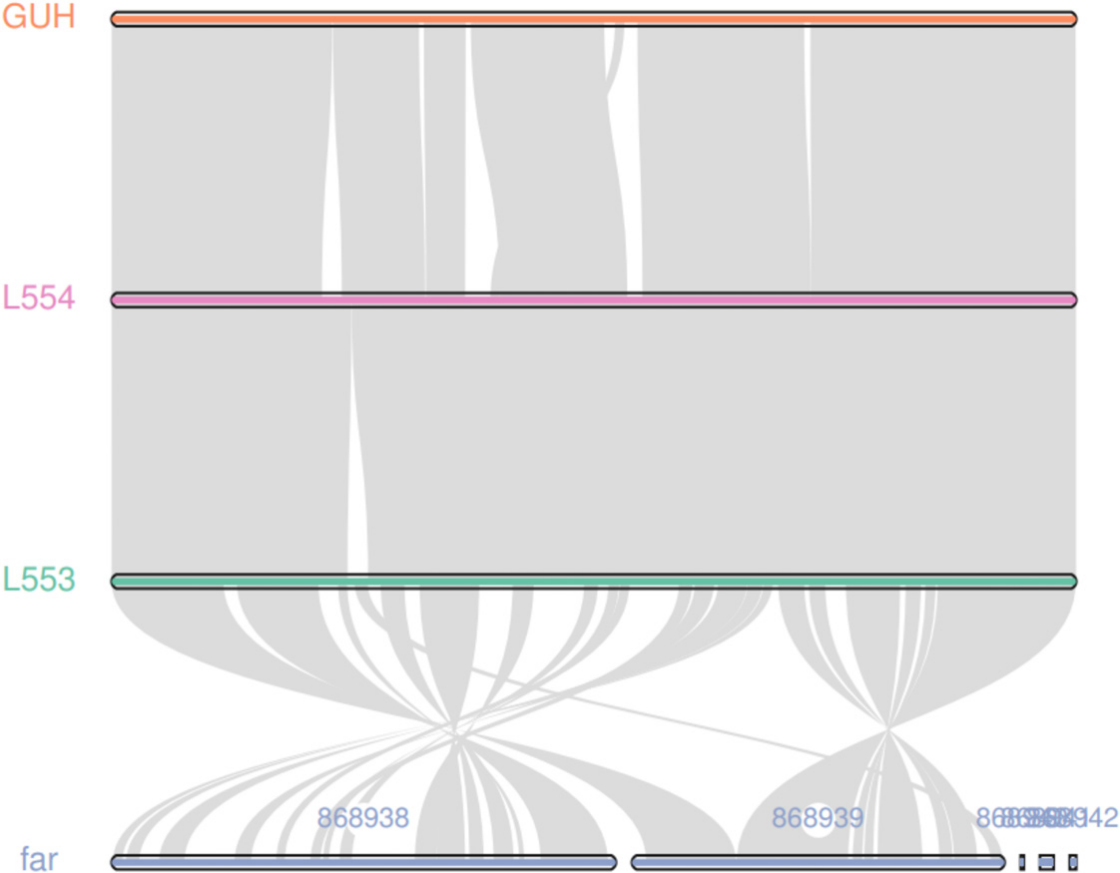
Colinear analysis of the coding gene sequence of strains L5.53 and L5.54. The coding genes of isolates L5.53 and L5.54 were highly consistent with *N. cyriacigeorgica* GUH-2, and isolate L5.53 and *N. farcinica* had many same collinear gene sequences. But there were lots of genomic rearrangements such as deletion, inversion and translocation between them.

The average sequencing quality scores for both strains of isolate L5.53 and L5.54 were 0.87, Q20 of two strains were more than 97%, Q30 were more than 93%, and the number of reads was 80,487 and 199,927, respectively. The data volume of isolate L5.53 was 648,949,509 bp, and the average sequencing read length was 8,063 bp. The data volume of isolate L5.54 was 1,428,886,617 bp, and the average sequencing read length was 7,147 bp. Isolate L5.53 had a genome size of 6,465,926bp with an in silico G + C content of 68.5%. Isolate L5.54 had a genome size of 6,387,902bp with an in silico G + C content of 68.33%. Both of L5.53 and L5.54 were assembled as circular sequences with N50 contig length of 6,478,940 bp and 6,395,880 bp respectively. As can be seen from the constructed phylogenetic tree (**Figure 3**), isolate L5.53 and L5. 54 were both *N. cyriacigeorgica*, forming a good supported subclade with *N. cyriacigeorgica* CNM20110624. Aligning their coding gene sequences, a total of 155 indels and 108 SNPs were counted, corresponding to 103 sequences of isolate L5.53 and 104 sequences of isolate L5.54, respectively. The results of the colinear alignment of the coding gene sequences were shown in **Figure 4**. The coding genes of isolates L5.53 and L5.54 were highly consistent with *N. cyriacigeorgica* GUH-2 (Beaman and Maslan, 1977). Details of these reference sequences in the gene family cluster analysis evolutionary tree were presented in **Table S1**.

By aligning the protein sequences of isolate L5.53 and L5.54, it was found that one of the sequences with differences was annotated as septum formation family protein (*N. cyriacigeorgica*) in the NCBI-nr database. In the nr database, 4858 genes of isolate L5.53 and 4861 genes of isolate L5.54 were annotated as *N. cyriacigeorgica*. The functions annotated in the GO database were mainly concentrated in enzyme regulation activities, catalytic activities, and metabolic processes. Most of the genes annotated in the KEGG database were related to metabolism, and genes related to human diseases were also annotated. The PHI database annotation results of isolate L5.53 and L5.54 showed that there were 330 genes in isolate L5.53 and 331 genes in isolate L5.54, many of which were genes of reduced virulence, also including genes of unaffected pathogenicity and increased virulence (hypervirulence). Isolate L5.53 and isolate L5.54 had the same number of genes of increased virulence, which was 21. The alignment to genes related to human diseases was also the same, including *Rv2626c*, *Rv1093* (*glyA1*) gene and *lon* gene.

The annotation results of isolate L5.53 and L5.54 in the ARDB database were the same, with 19 genes annotated, including 16 antibiotic resistance genes: *bcrA*, *vanRB*, *cml_e6*, *pur8*, *srmB*, *oleb*, *tcr3*, *bl2a_iii*, *vatE*, *vgbb*, *bacA*, *ermh*, *vanRC*, *aph3va*, *cml_e7*, and *dfra26*. In the alignment results of the CARD database, isolate L5.53 annotated 41 genes, of which the most annotated resistance gene was *lrfA*, a total of 12 genes aligned to it. The number of genes aligned to *drrC* was 4, the number of genes aligned for *embB* (*Mycobacterium bovis*), *TaeA*, and *drrB* was 2, and the number of *ileS* (*Bifidobacteria intrinsic*), *efpA*, *tet43*, *gyrA* (*M. tuberculosis*), *mfd*, *abcA*, *embB* (*M. tuberculosis*), *tetB*, *kasA* (*M. tuberculosis*), *tetA*, *katG* (*M. tuberculosis*), *mtrA*, *cmlv*, *murA* (*M. tuberculosis*), *gyrB* (*Escherichia coli*), *desR*, *alaS* and *qepA* was 1. The alignment results of isolate L5.54 were similar to those of L5.53, except that the number of genes aligned to *alaS* in L5.54 was 3, and the *carA* gene, which was not aligned to isolate L5.53, was aligned. Virulence factors and related gene products included *Capsule*, *Mce*, Pyrimidine biosynthesis, and WhiB3.

## Discussion

The same species in genus *Nocardia* may have different colony morphology, colony color and bacterial characteristics under the microscope, which may be an important reason why the bacterial morphology culture cannot accurately identify *Nocardia* spp. In this study, isolates L5.53 and L5.54 had significantly different morphological culture characteristics. However, after whole-genome resequencing and comparative genomic studies, both isolates L5.53 and L5.54 were classified as *N. cyriacigeorgica*. *N. cyriacigeorgica* was defined as a species in 2001 (Yassin et al., 2001), but it is not an emerging pathogen. At present, *N. cyriacigeorgica* is the main pathogenic species of nocardiosis in Japan, Thailand and Taiwan, and the reported rate has gradually increased in China mainland in recent years, reaching 46% in some areas (Quan et al., 2020). It is necessary to quickly and accurately identify *N. cyriacigeorgica*, but traditional methods, such as microbial isolation and culture identification, obviously cannot do.

The sequence annotated as septum formation family protein (*N. cyriacigeorgica*) is most likely responsible for the morphological difference between isolates L5.54 and L5.53. According to information in the NCBI-nr database, this domain is known to be present in a protein that is predicted to play a role in diaphragm formation during cell division. One study showed that obstruction of the diaphragm during cell division led to bacterial filamentation (Slayden et al., 2006). From the microscope morphology of the two samples, it can be seen that the shape of the isolate L5.53 was not straight, like the branches of a mulberry tree; the hyphae of the isolate L5.54 were stretched and filamentous. Therefore, the difference between the two homologous sequences of isolates L5.53 and L5.54 may be the potential reason for the difference in morphology between the two samples although they are of the same species by mass spectrometry (**Figure S1**).

In this study, isolates L5.53 and L5.54 had the same genes associated with human disease (*Rv2626c*, *Rv1093* (*glyA1*) and *lon* genes). *Rv2626c* and *Rv1093* (*glyA1*) genes are pathogenic genes of *M. tuberculosis*. Previous studies found that the *Rv2626c* gene and downstream gene expression could cause MTB to enter dormancy and evade the host immune system (Danelishvili et al., 2016). The *lon* gene is closely related to the pathogenicity of *Salmonella*. Lon protease is indispensable for *Salmonella* systemic infection and phagocytic survival during dissemination stages in the host (Kirthika et al., 2020). Therefore, the pathogenicity of isolates L5.53 and L5.54 may be similar to that of *M. tuberculosis* and *Salmonella* sp.

According to the resistance gene annotation results, a total of 40 resistance genes were found, which were divided into four categories according to the way each gene played a role in resistance. The most abundant resistance family found was the efflux pump, promoting the efflux of bactericidal substances such as chloramphenicol, macrolide-lincosamide-streptomycin B, tetracycline, bacitracin, etc., which also happened in other studies (Xue et al., 2021). The second type of resistance genes produced a variety of enzymes that made the antibacterial properties of the drug ineffective or inactivate the target drug, such as class A β-lactamase, Virginiamycin A acetyltransferase, streptococcus B enzyme, etc. The third class of resistance genes worked by producing resistance proteins, including *embB* (*M. tuberculosis*) and *alaS* (*polyamine-resistant proteins*), *gyrA* (fluoroquinolone resistance protein), *ileS*(mupirocin-resistant proteins), *embB* (*M. bovis*) (polyamine resistance protein), *katG* and *kasA* (isoniazid resistance protein), *Streptomyces cinnamoneus* EF-Tu (elfamycin resistance protein),and *gyrB* (aminocoumarin proteins). The last class conferred vancomycin, tetracycline and trimethoprim resistance to bacteria.

Isolates L5.53 and L5.54 contained three virulence factor-related genes, which were related to *Capsule*, WhiB3, Pyrimidine biosynthesis, and *Mce*. Capsule-related genes, such as *cpsI*, *uppS*, *cap8J*, and *rmlB*, are involved in the synthesis of capsular polysaccharides. Different capsular polysaccharides are composed of glucose, galactose, N-acetylglucosamine and sialic acid, among which sialic acid plays an important role in virulence (Tzeng et al., 2016). The pathogenic mechanism of WhiB3 is the interaction of WhiB3 with the C-terminal region of SigA (RpoV), a major sigma factor that activates the expression of virulence determinants (Steyn et al., 2002). Since the discovery of WhiB protein, it has received extensive attention due to its role in regulating the developmental process and virulence of mycobacterial biology (Cailiang et al., 2021), so the WhiB3 protein found in *N. cyriacigeorgica* is likely to be related to its virulence. The large subunit of carbamoyl phosphate synthase is a gene product related to pyrimidine biosynthesis. For example, *carB*, *carA* and *pyrB* encode the large and small subunits of carbamoyl phosphate synthase and aspartate carbamoyl transferase, respectively, which are required for the virulence of several pathogens, including *Salmonella* sp. and *E. coli* (Le Breton et al., 2013). The *mce*3E, *mce*4A, *mce*4C, *mce*4D, and *mce*5B were included in *Mce* genes, translating proteins associated with the invasion and long-term existence of *Mycobacteria* in macrophages (Hemati et al., 2019). Studies have found that the virulence factors of Mce family proteins were related to the virulence and pathogenic expression of *N. asteroids* (Patra et al., 2020). Since *N. cyriacigeorgica* and *N. asteroides* belong to the same genus, the virulence factors may work in a similar manner.

In contrast to previous studies, this work was the first to use resequencing technology to study the whole-genome sequences of two strains of *N. cyriacigeorgica* with significantly different morphology. In conclusion, this report suggests that whole genome resequencing can be used to help clinicians accurately diagnose the patients are infected by *Nocardia* spp., which have significantly different morphology. What’s more, it is also helpful to reveal the reasons for the differences of bacterial drug resistance, virulence factors and morphological phenotypes from the genetic level. However, this study has some limitations. Firstly, due to experimental manipulation and sequencing data quality, the genome sequences of isolates L5.53 and L5.54 may be incomplete. Secondly, the sequence difference between the two strains of bacteria was too small to exclude other reasons leading to the morphological difference. Finally, we only predicted pathogenic genes, drug resistance genes and virulence factor related genes using bioinformatics tools without lab experiments to confirm the functions. The interesting functions of the various genes identified in this study require further investigation.

## Data availability statement

The datasets presented in this study can be found in online repositories. The names of the repository/repositories and accession number(s) can be found below: https://db.cngb.org, CNP0002924.

## Author contributions

AL and XL collected samples, extracted nucleic acids, constructed libraries and wrote the first draft of the manuscript. YL, ZG and RT downloaded and analyzed the two *N. cyriacigeorgica* sequences under study, validated functions necessary for sequences analyses and performed the computations. YH made a formal analysis. MH, ZF and LZ reviewed the final version of the manuscript, designed and supervised the study, reviewed and edited the manuscript, funding application, and project administration. All authors contributed to the article and approved the submitted version.

## Funding

This research was funded by Key Research and Development Projects of Science and Technology Department of Sichuan Province (Grant No. 2019YFS0319) and Sansure Biotech Transfusion Medicine Development Fund of Chinese Society of Blood Transfusion (CSBT-SX-2021-01).

## Acknowledgments

We are grateful for the support of the healthcare workers at Hebei Chest Hospital and appreciate all patients who volunteered to participate in this study.

## Conflict of interest

The authors declare that the research was conducted in the absence of any commercial or financial relationships that could be construed as a potential conflict of interest.

## Publisher’s note

All claims expressed in this article are solely those of the authors and do not necessarily represent those of their affiliated organizations, or those of the publisher, the editors and the reviewers. Any product that may be evaluated in this article, or claim that may be made by its manufacturer, is not guaranteed or endorsed by the publisher.
