# Supplementary material for: Two chronically misdiagnosed patients infected with Nocardia cyriacigeorgica accurately diagnosed by whole genome resequencing
Source: Front Cell Infect Microbiol. 2022 Oct 12;12:1032669. doi: 10.3389/fcimb.2022.1032669 (PMC9601747; doi:10.3389/fcimb.2022.1032669)
Supplement: Supplementary file 1 [file DataSheet_2.pdf]

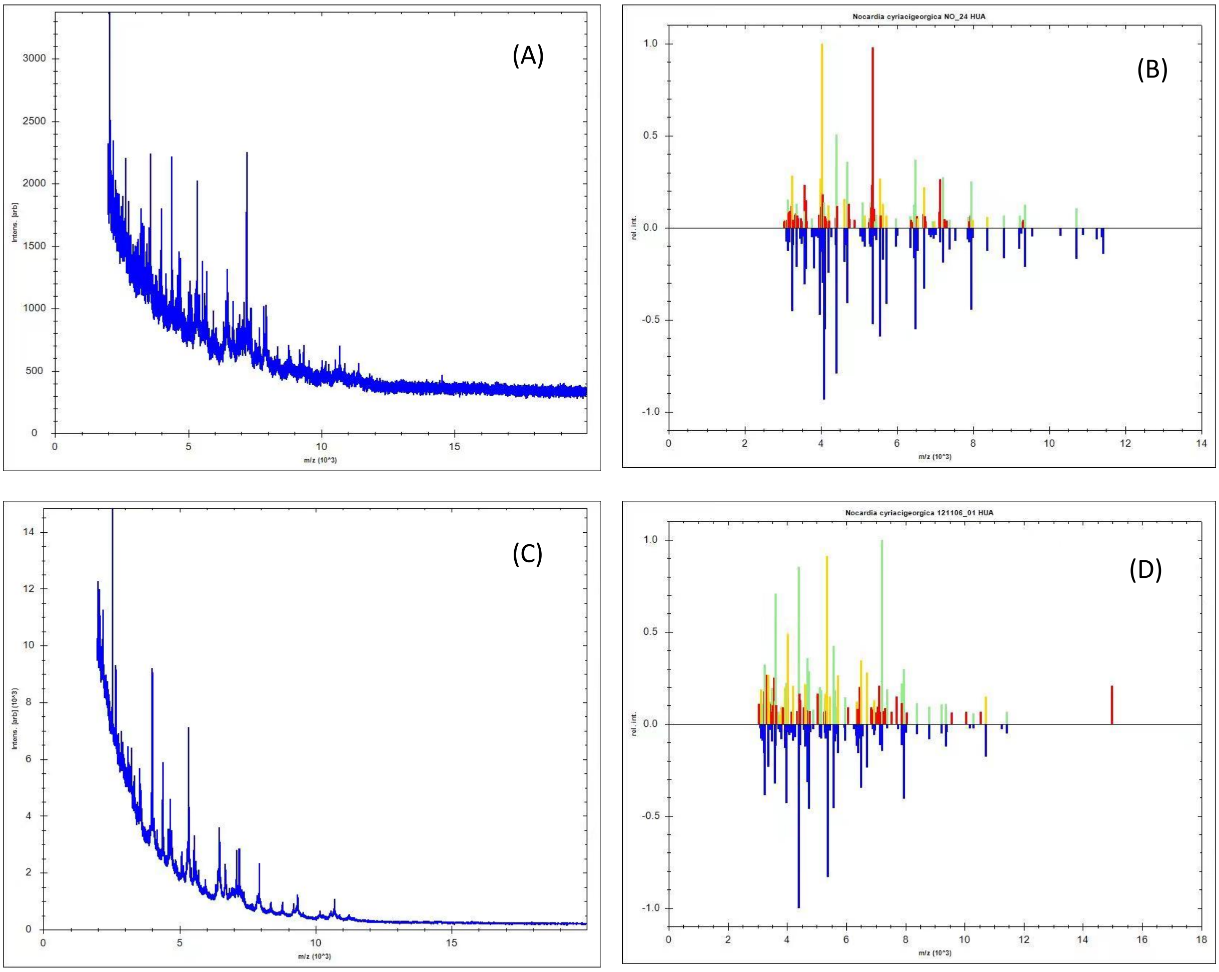

Supplementary Figure 1. Mass spectrometry results of L5.53 and L5.54 which were identified as *Nocardia* spp.
