# Supplementary material for: Two chronically misdiagnosed patients infected with Nocardia cyriacigeorgica accurately diagnosed by whole genome resequencing
Source: Front Cell Infect Microbiol. 2022 Oct 12;12:1032669. doi: 10.3389/fcimb.2022.1032669 (PMC9601747; doi:10.3389/fcimb.2022.1032669)
Supplement: Supplementary file 2 [file DataSheet_3.docx]

Supplementary Material

Table S1. Details of reference sequences in the gene family cluster analysis evolutionary tree.

| **Species** | **ID** | **Description**  **(Refer to CGMCC）** | **Protein** | **Genome file (representative genome)** | **GFF** | **CDS** | **Description** | **Owner** | **Details** |  |
| --- | --- | --- | --- | --- | --- | --- | --- | --- | --- | --- |
| *Nocardia_cyriacigeorgica*_MDA3349 | 135487 | *Nocardia_cyri-acigeorgica* | ftp://ftp.ncbi.nlm.nih.gov/genomes/all/GCF/002/949/635/GCF_002949635.1_ASM294963v1/GCF_002949635.1_ASM294963v1_protein.faa.gz | ftp://ftp.ncbi.nlm.nih.gov/genomes/all/GCF/002/949/635/GCF_002949635.1_ASM294963v1/GCF_002949635.1_ASM294963v1_genomic.fna.gz | ftp://ftp.ncbi.nlm.nih.gov/genomes/all/GCF/002/949/635/GCF_002949635.1_ASM294963v1/GCF_002949635.1_ASM294963v1_genomic.gff.gz | ftp://ftp.ncbi.nlm.nih.gov/genomes/all/GCF/002/949/635/GCF_002949635.1_ASM294963v1/GCF_002949635.1_ASM294963v1_cds_from_genomic.fna.gz | Pathogen: clinical or host-associated sample from *Nocardia cyriacigeorgica* | Rice University | https://www.ncbi.nlm.nih.gov/data-hub/assembly/GCF_002949635.1/ |  |
| *Nocardia_cyriacigeorgica*_3012STDY6756504 | 135487 | *Nocardia_cyri-acigeorgica* | ftp://ftp.ncbi.nlm.nih.gov/genomes/all/GCF/900/683/635/GCF_900683635.1_54424_D01/GCF_900683635.1_54424_D01_protein.faa.gz | ftp://ftp.ncbi.nlm.nih.gov/genomes/all/GCF/900/683/635/GCF_900683635.1_54424_D01/GCF_900683635.1_54424_D01_genomic.fna.gz | ftp://ftp.ncbi.nlm.nih.gov/genomes/all/GCF/900/683/635/GCF_900683635.1_54424_D01/GCF_900683635.1_54424_D01_genomic.gff.gz | ftp://ftp.ncbi.nlm.nih.gov/genomes/all/GCF/900/683/635/GCF_900683635.1_54424_D01/GCF_900683635.1_54424_D01_cds_from_genomic.fna.gz | 54424_D01 | EBI | https://www.ncbi.nlm.nih.gov/data-hub/assembly/GCF_900683635.1/ |  |
| *Nocardia_cyriacigeorgica*_GUH-2 | 135487 | *Nocardia_cyri-acigeorgica* | <ftp://ftp.ncbi.nlm.nih.gov/genomes/all/GCF/000/284/035/GCF_000284035.1_ASM28403v1/GCF_000284035.1_ASM28403v1_protein.faa.gz> | ftp://ftp.ncbi.nlm.nih.gov/genomes/all/GCF/000/284/035/GCF_000284035.1_ASM28403v1/GCF_000284035.1_ASM28403v1_genomic.fna.gz | ftp://ftp.ncbi.nlm.nih.gov/genomes/all/GCF/000/284/035/GCF_000284035.1_ASM28403v1/GCF_000284035.1_ASM28403v1_genomic.gff.gz | ftp://ftp.ncbi.nlm.nih.gov/genomes/all/GCF/000/284/035/GCF_000284035.1_ASM28403v1/GCF_000284035.1_ASM28403v1_cds_from_genomic.fna.gz | BioSample entry for genome collection GCA_000284035 | EBI | https://www.ncbi.nlm.nih.gov/data-hub/assembly/GCF_000284035.1/ |  |
| *Nocardia_cyriacigeorgica*_DSM_44484 | 135487 | *Nocardia_cyri-acigeorgica* | <ftp://ftp.ncbi.nlm.nih.gov/genomes/all/GCF/005/863/225/GCF_005863225.1_ASM586322v1/GCF_005863225.1_ASM586322v1_protein.faa.gz> | ftp://ftp.ncbi.nlm.nih.gov/genomes/all/GCF/005/863/225/GCF_005863225.1_ASM586322v1/GCF_005863225.1_ASM586322v1_genomic.fna.gz | ftp://ftp.ncbi.nlm.nih.gov/genomes/all/GCF/005/863/225/GCF_005863225.1_ASM586322v1/GCF_005863225.1_ASM586322v1_genomic.gff.gz | ftp://ftp.ncbi.nlm.nih.gov/genomes/all/GCF/005/863/225/GCF_005863225.1_ASM586322v1/GCF_005863225.1_ASM586322v1_cds_from_genomic.fna.gz | Microbe sample from *Nocardia cyriacigeorgica* | UMR5557 Laboratoire d'Ecologie Microbienne | https://www.ncbi.nlm.nih.gov/data-hub/assembly/GCF_005863225.1/ |  |
| *Nocardia_cyriacigeorgica*_NBRC_100375 | 135487 | *Nocardia_cyri-acigeorgica* | ftp://ftp.ncbi.nlm.nih.gov/genomes/all/GCF/000/308/555/GCF_000308555.1_ASM30855v1/GCF_000308555.1_ASM30855v1_protein.faa.gz | ftp://ftp.ncbi.nlm.nih.gov/genomes/all/GCF/000/308/555/GCF_000308555.1_ASM30855v1/GCF_000308555.1_ASM30855v1_genomic.fna.gz | ftp://ftp.ncbi.nlm.nih.gov/genomes/all/GCF/000/308/555/GCF_000308555.1_ASM30855v1/GCF_000308555.1_ASM30855v1_genomic.gff.gz | ftp://ftp.ncbi.nlm.nih.gov/genomes/all/GCF/000/308/555/GCF_000308555.1_ASM30855v1/GCF_000308555.1_ASM30855v1_cds_from_genomic.fna.gz | Generic sample from *Nocardia cyriacigeorgica* NBRC 100375 | National Institute of Technology and Evaluation Biological Resource Center | https://www.ncbi.nlm.nih.gov/data-hub/assembly/GCF_000308555.1/ |  |
| *Nocardia_cyriacigeorgica*_BJ06-0127 | 135487 | *Nocardia_cyri-acigeorgica* | ftp://ftp.ncbi.nlm.nih.gov/genomes/all/GCF/015/478/075/GCF_015478075.1_ASM1547807v1/GCF_015478075.1_ASM1547807v1_protein.faa.gz | ftp://ftp.ncbi.nlm.nih.gov/genomes/all/GCF/015/478/075/GCF_015478075.1_ASM1547807v1/GCF_015478075.1_ASM1547807v1_genomic.fna.gz | ftp://ftp.ncbi.nlm.nih.gov/genomes/all/GCF/015/478/075/GCF_015478075.1_ASM1547807v1/GCF_015478075.1_ASM1547807v1_genomic.gff.gz | ftp://ftp.ncbi.nlm.nih.gov/genomes/all/GCF/015/478/075/GCF_015478075.1_ASM1547807v1/GCF_015478075.1_ASM1547807v1_cds_from_genomic.fna.gz | Microbe sample from *Nocardia cyriacigeorgica* | Chinese PLA Center for Disease Control and Prevention | https://www.ncbi.nlm.nih.gov/data-hub/assembly/GCA_015478075.1/ |  |
| *Nocardia_cyriacigeorgica*_BJ06-0062 | 135487 | *Nocardia_cyri-acigeorgica* | ftp://ftp.ncbi.nlm.nih.gov/genomes/all/GCF/015/478/435/GCF_015478435.1_ASM1547843v1/GCF_015478435.1_ASM1547843v1_protein.faa.gz | ftp://ftp.ncbi.nlm.nih.gov/genomes/all/GCF/015/478/435/GCF_015478435.1_ASM1547843v1/GCF_015478435.1_ASM1547843v1_genomic.fna.gz | ftp://ftp.ncbi.nlm.nih.gov/genomes/all/GCF/015/478/435/GCF_015478435.1_ASM1547843v1/GCF_015478435.1_ASM1547843v1_genomic.gff.gz | ftp://ftp.ncbi.nlm.nih.gov/genomes/all/GCF/015/478/435/GCF_015478435.1_ASM1547843v1/GCF_015478435.1_ASM1547843v1_cds_from_genomic.fna.gz | Microbe sample from *Nocardia cyriacigeorgica* | Chinese PLA Center for Disease Control and Prevention | https://www.ncbi.nlm.nih.gov/data-hub/assembly/GCA_015478435.1/ |  |
| *Nocardia_cyriacigeorgica*_N-26 | 135487 | *Nocardia_cyri-acigeorgica* | ftp://ftp.ncbi.nlm.nih.gov/genomes/all/GCF/015/477/225/GCF_015477225.1_ASM1547722v1/GCF_015477225.1_ASM1547722v1_protein.faa.gz | ftp://ftp.ncbi.nlm.nih.gov/genomes/all/GCF/015/477/225/GCF_015477225.1_ASM1547722v1/GCF_015477225.1_ASM1547722v1_genomic.fna.gz | ftp://ftp.ncbi.nlm.nih.gov/genomes/all/GCF/015/477/225/GCF_015477225.1_ASM1547722v1/GCF_015477225.1_ASM1547722v1_genomic.gff.gz | ftp://ftp.ncbi.nlm.nih.gov/genomes/all/GCF/015/477/225/GCF_015477225.1_ASM1547722v1/GCF_015477225.1_ASM1547722v1_cds_from_genomic.fna.gz | Chinese PLA Center for Disease Control and Prevention | Microbe sample from *Nocardia cyriacigeorgica* | https://www.ncbi.nlm.nih.gov/data-hub/assembly/GCF_015477225.1/ |  |
| *Nocardia_cyriacigeorgica*_BJ06-0121 | 135487 | *Nocardia_cyri-acigeorgica* | ftp://ftp.ncbi.nlm.nih.gov/genomes/all/GCF/015/478/165/GCF_015478165.1_ASM1547816v1/GCF_015478165.1_ASM1547816v1_protein.faa.gz | ftp://ftp.ncbi.nlm.nih.gov/genomes/all/GCF/015/478/165/GCF_015478165.1_ASM1547816v1/GCF_015478165.1_ASM1547816v1_genomic.fna.gz | ftp://ftp.ncbi.nlm.nih.gov/genomes/all/GCF/015/478/165/GCF_015478165.1_ASM1547816v1/GCF_015478165.1_ASM1547816v1_genomic.gff.gz | ftp://ftp.ncbi.nlm.nih.gov/genomes/all/GCF/015/478/165/GCF_015478165.1_ASM1547816v1/GCF_015478165.1_ASM1547816v1_cds_from_genomic.fna.gz | Microbe sample from *Nocardia cyriacigeorgica* | Chinese PLA Center for Disease Control and Prevention | https://www.ncbi.nlm.nih.gov/data-hub/assembly/GCF_015478165.1/ |  |
| *Nocardia_cyriacigeorgica*_BJ06-0134 | 135487 | *Nocardia_cyri-acigeorgica* | ftp://ftp.ncbi.nlm.nih.gov/genomes/all/GCF/015/477/945/GCF_015477945.1_ASM1547794v1/GCF_015477945.1_ASM1547794v1_protein.faa.gz | ftp://ftp.ncbi.nlm.nih.gov/genomes/all/GCF/015/477/945/GCF_015477945.1_ASM1547794v1/GCF_015477945.1_ASM1547794v1_genomic.fna.gz | ftp://ftp.ncbi.nlm.nih.gov/genomes/all/GCF/015/477/945/GCF_015477945.1_ASM1547794v1/GCF_015477945.1_ASM1547794v1_genomic.gff.gz | ftp://ftp.ncbi.nlm.nih.gov/genomes/all/GCF/015/477/945/GCF_015477945.1_ASM1547794v1/GCF_015477945.1_ASM1547794v1_cds_from_genomic.fna.gz | Microbe sample from *Nocardia cyriacigeorgica* | Chinese PLA Center for Disease Control and Prevention | https://www.ncbi.nlm.nih.gov/data-hub/assembly/GCA_015477945.1/ |  |
| *Nocardia_cyriacigeorgica*_BJ06-0130 | 135487 | *Nocardia_cyri-acigeorgica* | ftp://ftp.ncbi.nlm.nih.gov/genomes/all/GCF/015/478/055/GCF_015478055.1_ASM1547805v1/GCF_015478055.1_ASM1547805v1_protein.faa.gz | ftp://ftp.ncbi.nlm.nih.gov/genomes/all/GCF/015/478/055/GCF_015478055.1_ASM1547805v1/GCF_015478055.1_ASM1547805v1_genomic.fna.gz | ftp://ftp.ncbi.nlm.nih.gov/genomes/all/GCF/015/478/055/GCF_015478055.1_ASM1547805v1/GCF_015478055.1_ASM1547805v1_genomic.gff.gz | ftp://ftp.ncbi.nlm.nih.gov/genomes/all/GCF/015/478/055/GCF_015478055.1_ASM1547805v1/GCF_015478055.1_ASM1547805v1_cds_from_genomic.fna.gz | Microbe sample from *Nocardia cyriacigeorgica* | Chinese PLA Center for Disease Control and Prevention | https://www.ncbi.nlm.nih.gov/data-hub/assembly/GCA_015478055.1/ |  |
| *Nocardia_cyriacigeorgica*_BJ06-0149 | 135487 | *Nocardia_cyri-acigeorgica* | ftp://ftp.ncbi.nlm.nih.gov/genomes/all/GCF/015/477/725/GCF_015477725.1_ASM1547772v1/GCF_015477725.1_ASM1547772v1_protein.faa.gz | ftp://ftp.ncbi.nlm.nih.gov/genomes/all/GCF/015/477/725/GCF_015477725.1_ASM1547772v1/GCF_015477725.1_ASM1547772v1_genomic.fna.gz | ftp://ftp.ncbi.nlm.nih.gov/genomes/all/GCF/015/477/725/GCF_015477725.1_ASM1547772v1/GCF_015477725.1_ASM1547772v1_genomic.gff.gz | ftp://ftp.ncbi.nlm.nih.gov/genomes/all/GCF/015/477/725/GCF_015477725.1_ASM1547772v1/GCF_015477725.1_ASM1547772v1_cds_from_genomic.fna.gz | Microbe sample from *Nocardia cyriacigeorgica* | Chinese PLA Center for Disease Control and Prevention | https://www.ncbi.nlm.nih.gov/data-hub/assembly/GCF_015477725.1/ |  |
| *Nocardia_cyriacigeorgica*_N-43 | 135487 | *Nocardia_cyri-acigeorgica* | ftp://ftp.ncbi.nlm.nih.gov/genomes/all/GCF/015/476/925/GCF_015476925.1_ASM1547692v1/GCF_015476925.1_ASM1547692v1_protein.faa.gz | ftp://ftp.ncbi.nlm.nih.gov/genomes/all/GCF/015/476/925/GCF_015476925.1_ASM1547692v1/GCF_015476925.1_ASM1547692v1_genomic.fna.gz | ftp://ftp.ncbi.nlm.nih.gov/genomes/all/GCF/015/476/925/GCF_015476925.1_ASM1547692v1/GCF_015476925.1_ASM1547692v1_genomic.gff.gz | ftp://ftp.ncbi.nlm.nih.gov/genomes/all/GCF/015/476/925/GCF_015476925.1_ASM1547692v1/GCF_015476925.1_ASM1547692v1_cds_from_genomic.fna.gz | Microbe sample from *Nocardia cyriacigeorgica* | Chinese PLA Center for Disease Control and Prevention | https://www.ncbi.nlm.nih.gov/data-hub/assembly/GCA_015476925.1/ |  |
| *Nocardia_cyriacigeorgica*_N-48 | 135487 | *Nocardia_cyri-acigeorgica* | ftp://ftp.ncbi.nlm.nih.gov/genomes/all/GCF/015/476/905/GCF_015476905.1_ASM1547690v1/GCF_015476905.1_ASM1547690v1_protein.faa.gz | ftp://ftp.ncbi.nlm.nih.gov/genomes/all/GCF/015/476/905/GCF_015476905.1_ASM1547690v1/GCF_015476905.1_ASM1547690v1_genomic.fna.gz | ftp://ftp.ncbi.nlm.nih.gov/genomes/all/GCF/015/476/905/GCF_015476905.1_ASM1547690v1/GCF_015476905.1_ASM1547690v1_genomic.gff.gz | ftp://ftp.ncbi.nlm.nih.gov/genomes/all/GCF/015/476/905/GCF_015476905.1_ASM1547690v1/GCF_015476905.1_ASM1547690v1_cds_from_genomic.fna.gz | Microbe sample from *Nocardia cyriacigeorgica* | Chinese PLA Center for Disease Control and Prevention | https://www.ncbi.nlm.nih.gov/data-hub/assembly/GCA_015476905.1/ |  |
| *Nocardia_cyriacigeorgica*_BJ06-0132 | 135487 | *Nocardia_cyri-acigeorgica* | ftp://ftp.ncbi.nlm.nih.gov/genomes/all/GCF/015/477/975/GCF_015477975.1_ASM1547797v1/GCF_015477975.1_ASM1547797v1_protein.faa.gz | ftp://ftp.ncbi.nlm.nih.gov/genomes/all/GCF/015/477/975/GCF_015477975.1_ASM1547797v1/GCF_015477975.1_ASM1547797v1_genomic.fna.gz | ftp://ftp.ncbi.nlm.nih.gov/genomes/all/GCF/015/477/975/GCF_015477975.1_ASM1547797v1/GCF_015477975.1_ASM1547797v1_genomic.gff.gz | ftp://ftp.ncbi.nlm.nih.gov/genomes/all/GCF/015/477/975/GCF_015477975.1_ASM1547797v1/GCF_015477975.1_ASM1547797v1_cds_from_genomic.fna.gz | Microbe sample from *Nocardia cyriacigeorgica* | Chinese PLA Center for Disease Control and Prevention | https://www.ncbi.nlm.nih.gov/data-hub/assembly/GCF_015477975.1/ |  |
| *Nocardia_cyriacigeorgica*_BJ06-0133 | 135487 | *Nocardia_cyri-acigeorgica* | ftp://ftp.ncbi.nlm.nih.gov/genomes/all/GCF/015/477/995/GCF_015477995.1_ASM1547799v1/GCF_015477995.1_ASM1547799v1_protein.faa.gz | ftp://ftp.ncbi.nlm.nih.gov/genomes/all/GCF/015/477/995/GCF_015477995.1_ASM1547799v1/GCF_015477995.1_ASM1547799v1_genomic.fna.gz | ftp://ftp.ncbi.nlm.nih.gov/genomes/all/GCF/015/477/995/GCF_015477995.1_ASM1547799v1/GCF_015477995.1_ASM1547799v1_genomic.gff.gz | ftp://ftp.ncbi.nlm.nih.gov/genomes/all/GCF/015/477/995/GCF_015477995.1_ASM1547799v1/GCF_015477995.1_ASM1547799v1_cds_from_genomic.fna.gz | Microbe sample from *Nocardia cyriacigeorgica* | Chinese PLA Center for Disease Control and Prevention | https://www.ncbi.nlm.nih.gov/data-hub/assembly/GCF_015477995.1/ |  |
| *Nocardia_cyriacigeorgica*_EML_446 | 135487 | *Nocardia_cyri-acigeorgica* | ftp://ftp.ncbi.nlm.nih.gov/genomes/all/GCF/005/863/245/GCF_005863245.1_ASM586324v1/GCF_005863245.1_ASM586324v1_protein.faa.gz | ftp://ftp.ncbi.nlm.nih.gov/genomes/all/GCF/005/863/245/GCF_005863245.1_ASM586324v1/GCF_005863245.1_ASM586324v1_genomic.fna.gz | ftp://ftp.ncbi.nlm.nih.gov/genomes/all/GCF/005/863/245/GCF_005863245.1_ASM586324v1/GCF_005863245.1_ASM586324v1_genomic.gff.gz | ftp://ftp.ncbi.nlm.nih.gov/genomes/all/GCF/005/863/245/GCF_005863245.1_ASM586324v1/GCF_005863245.1_ASM586324v1_cds_from_genomic.fna.gz | Microbe sample from *Nocardia cyriacigeorgica* | UMR5557 Laboratoire d'Ecologie Microbienne | https://www.ncbi.nlm.nih.gov/data-hub/assembly/GCA_005863245.1/ |  |
| *Nocardia_cyriacigeorgica*_BJ06-0147 | 135487 | *Nocardia_cyri-acigeorgica* | ftp://ftp.ncbi.nlm.nih.gov/genomes/all/GCF/015/477/705/GCF_015477705.1_ASM1547770v1/GCF_015477705.1_ASM1547770v1_protein.faa.gz | ftp://ftp.ncbi.nlm.nih.gov/genomes/all/GCF/015/477/705/GCF_015477705.1_ASM1547770v1/GCF_015477705.1_ASM1547770v1_genomic.fna.gz | ftp://ftp.ncbi.nlm.nih.gov/genomes/all/GCF/015/477/705/GCF_015477705.1_ASM1547770v1/GCF_015477705.1_ASM1547770v1_genomic.gff.gz | ftp://ftp.ncbi.nlm.nih.gov/genomes/all/GCF/015/477/705/GCF_015477705.1_ASM1547770v1/GCF_015477705.1_ASM1547770v1_cds_from_genomic.fna.gz | Microbe sample from *Nocardia cyriacigeorgica* | Chinese PLA Center for Disease Control and Prevention | https://www.ncbi.nlm.nih.gov/data-hub/assembly/GCA_015477705.1/ |  |
| *Nocardia_cyriacigeorgica*_N-32 | 135487 | *Nocardia_cyri-acigeorgica* | ftp://ftp.ncbi.nlm.nih.gov/genomes/all/GCF/015/477/145/GCF_015477145.1_ASM1547714v1/GCF_015477145.1_ASM1547714v1_protein.faa.gz | ftp://ftp.ncbi.nlm.nih.gov/genomes/all/GCF/015/477/145/GCF_015477145.1_ASM1547714v1/GCF_015477145.1_ASM1547714v1_genomic.fna.gz | ftp://ftp.ncbi.nlm.nih.gov/genomes/all/GCF/015/477/145/GCF_015477145.1_ASM1547714v1/GCF_015477145.1_ASM1547714v1_genomic.gff.gz | ftp://ftp.ncbi.nlm.nih.gov/genomes/all/GCF/015/477/145/GCF_015477145.1_ASM1547714v1/GCF_015477145.1_ASM1547714v1_cds_from_genomic.fna.gz | Microbe sample from *Nocardia cyriacigeorgica* | Chinese PLA Center for Disease Control and Prevention | https://www.ncbi.nlm.nih.gov/data-hub/assembly/GCF_015477145.1/ |  |
| *Nocardia_cyriacigeorgica*_N-18 | 135487 | *Nocardia_cyri-acigeorgica* | ftp://ftp.ncbi.nlm.nih.gov/genomes/all/GCF/015/477/345/GCF_015477345.1_ASM1547734v1/GCF_015477345.1_ASM1547734v1_protein.faa.gz | ftp://ftp.ncbi.nlm.nih.gov/genomes/all/GCF/015/477/345/GCF_015477345.1_ASM1547734v1/GCF_015477345.1_ASM1547734v1_genomic.fna.gz | ftp://ftp.ncbi.nlm.nih.gov/genomes/all/GCF/015/477/345/GCF_015477345.1_ASM1547734v1/GCF_015477345.1_ASM1547734v1_genomic.gff.gz | ftp://ftp.ncbi.nlm.nih.gov/genomes/all/GCF/015/477/345/GCF_015477345.1_ASM1547734v1/GCF_015477345.1_ASM1547734v1_cds_from_genomic.fna.gz | Microbe sample from *Nocardia cyriacigeorgica* | Chinese PLA Center for Disease Control and Prevention | https://www.ncbi.nlm.nih.gov/data-hub/assembly/GCF_015477345.1/ |  |
| *Nocardia_cyriacigeorgica*_BJ06-0097 | 135487 | *Nocardia_cyri-acigeorgica* | ftp://ftp.ncbi.nlm.nih.gov/genomes/all/GCF/015/478/375/GCF_015478375.1_ASM1547837v1/GCF_015478375.1_ASM1547837v1_protein.faa.gz | ftp://ftp.ncbi.nlm.nih.gov/genomes/all/GCF/015/478/375/GCF_015478375.1_ASM1547837v1/GCF_015478375.1_ASM1547837v1_genomic.fna.gz | ftp://ftp.ncbi.nlm.nih.gov/genomes/all/GCF/015/478/375/GCF_015478375.1_ASM1547837v1/GCF_015478375.1_ASM1547837v1_genomic.gff.gz | ftp://ftp.ncbi.nlm.nih.gov/genomes/all/GCF/015/478/375/GCF_015478375.1_ASM1547837v1/GCF_015478375.1_ASM1547837v1_cds_from_genomic.fna.gz | Microbe sample from *Nocardia cyriacigeorgica* | Chinese PLA Center for Disease Control and Prevention | https://www.ncbi.nlm.nih.gov/data-hub/assembly/GCF_015478375.1/ |  |
| *Nocardia_cyriacigeorgica*_BJ06-0142 | 135487 | *Nocardia_cyri-acigeorgica* | ftp://ftp.ncbi.nlm.nih.gov/genomes/all/GCF/015/477/865/GCF_015477865.1_ASM1547786v1/GCF_015477865.1_ASM1547786v1_protein.faa.gz | ftp://ftp.ncbi.nlm.nih.gov/genomes/all/GCF/015/477/865/GCF_015477865.1_ASM1547786v1/GCF_015477865.1_ASM1547786v1_genomic.fna.gz | ftp://ftp.ncbi.nlm.nih.gov/genomes/all/GCF/015/477/865/GCF_015477865.1_ASM1547786v1/GCF_015477865.1_ASM1547786v1_genomic.gff.gz | ftp://ftp.ncbi.nlm.nih.gov/genomes/all/GCF/015/477/865/GCF_015477865.1_ASM1547786v1/GCF_015477865.1_ASM1547786v1_cds_from_genomic.fna.gz | Microbe sample from *Nocardia cyriacigeorgica* | Chinese PLA Center for Disease Control and Prevention | https://www.ncbi.nlm.nih.gov/data-hub/assembly/GCA_015477865.1/ |  |
| *Nocardia_cyriacigeorgica*_BJ06-0071 | 135487 | *Nocardia_cyri-acigeorgica* | ftp://ftp.ncbi.nlm.nih.gov/genomes/all/GCF/015/478/465/GCF_015478465.1_ASM1547846v1/GCF_015478465.1_ASM1547846v1_protein.faa.gz | ftp://ftp.ncbi.nlm.nih.gov/genomes/all/GCF/015/478/465/GCF_015478465.1_ASM1547846v1/GCF_015478465.1_ASM1547846v1_genomic.fna.gz | ftp://ftp.ncbi.nlm.nih.gov/genomes/all/GCF/015/478/465/GCF_015478465.1_ASM1547846v1/GCF_015478465.1_ASM1547846v1_genomic.gff.gz | ftp://ftp.ncbi.nlm.nih.gov/genomes/all/GCF/015/478/465/GCF_015478465.1_ASM1547846v1/GCF_015478465.1_ASM1547846v1_cds_from_genomic.fna.gz | Microbe sample from *Nocardia cyriacigeorgica* | Chinese PLA Center for Disease Control and Prevention | https://www.ncbi.nlm.nih.gov/data-hub/assembly/GCA_015478465.1/ |  |
| *Nocardia_cyriacigeorgica*_N-51 | 135487 | *Nocardia_cyri-acigeorgica* | ftp://ftp.ncbi.nlm.nih.gov/genomes/all/GCF/015/476/895/GCF_015476895.1_ASM1547689v1/GCF_015476895.1_ASM1547689v1_protein.faa.gz | ftp://ftp.ncbi.nlm.nih.gov/genomes/all/GCF/015/476/895/GCF_015476895.1_ASM1547689v1/GCF_015476895.1_ASM1547689v1_genomic.fna.gz | ftp://ftp.ncbi.nlm.nih.gov/genomes/all/GCF/015/476/895/GCF_015476895.1_ASM1547689v1/GCF_015476895.1_ASM1547689v1_genomic.gff.gz | ftp://ftp.ncbi.nlm.nih.gov/genomes/all/GCF/015/476/895/GCF_015476895.1_ASM1547689v1/GCF_015476895.1_ASM1547689v1_cds_from_genomic.fna.gz | Microbe sample from *Nocardia cyriacigeorgica* | Chinese PLA Center for Disease Control and Prevention | https://www.ncbi.nlm.nih.gov/data-hub/assembly/GCF_015478255.1/ |  |
| *Nocardia_cyriacigeorgica*_BJ06-0109 | 135487 | *Nocardia_cyri-acigeorgica* | ftp://ftp.ncbi.nlm.nih.gov/genomes/all/GCF/015/478/255/GCF_015478255.1_ASM1547825v1/GCF_015478255.1_ASM1547825v1_protein.faa.gz | ftp://ftp.ncbi.nlm.nih.gov/genomes/all/GCF/015/478/255/GCF_015478255.1_ASM1547825v1/GCF_015478255.1_ASM1547825v1_genomic.fna.gz | ftp://ftp.ncbi.nlm.nih.gov/genomes/all/GCF/015/478/255/GCF_015478255.1_ASM1547825v1/GCF_015478255.1_ASM1547825v1_genomic.gff.gz | ftp://ftp.ncbi.nlm.nih.gov/genomes/all/GCF/015/478/255/GCF_015478255.1_ASM1547825v1/GCF_015478255.1_ASM1547825v1_cds_from_genomic.fna.gz | Microbe sample from *Nocardia cyriacigeorgica* | Chinese PLA Center for Disease Control and Prevention | https://www.ncbi.nlm.nih.gov/data-hub/assembly/GCF_015476895.1/ |  |
| *Nocardia_cyriacigeorgica*_CNM20110629 | 135487 | *Nocardia_cyri-acigeorgica* | ftp://ftp.ncbi.nlm.nih.gov/genomes/all/GCF/010/857/985/GCF_010857985.1_ASM1085798v1/GCF_010857985.1_ASM1085798v1_protein.faa.gz | ftp://ftp.ncbi.nlm.nih.gov/genomes/all/GCF/010/857/985/GCF_010857985.1_ASM1085798v1/GCF_010857985.1_ASM1085798v1_genomic.fna.gz | ftp://ftp.ncbi.nlm.nih.gov/genomes/all/GCF/010/857/985/GCF_010857985.1_ASM1085798v1/GCF_010857985.1_ASM1085798v1_genomic.gff.gz | ftp://ftp.ncbi.nlm.nih.gov/genomes/all/GCF/010/857/985/GCF_010857985.1_ASM1085798v1/GCF_010857985.1_ASM1085798v1_cds_from_genomic.fna.gz | *Nocardia cyriacigeorgica* strain from soil | Instituto de Salud Carlos III | https://www.ncbi.nlm.nih.gov/data-hub/assembly/GCA_010857985.1/ |  |
| *Nocardia_cyriacigeorgica*_MDA3732 | 135487 | *Nocardia_cyri-acigeorgica* | ftp://ftp.ncbi.nlm.nih.gov/genomes/all/GCF/002/933/455/GCF_002933455.1_ASM293345v1/GCF_002933455.1_ASM293345v1_protein.faa.gz | ftp://ftp.ncbi.nlm.nih.gov/genomes/all/GCF/002/933/455/GCF_002933455.1_ASM293345v1/GCF_002933455.1_ASM293345v1_genomic.fna.gz | ftp://ftp.ncbi.nlm.nih.gov/genomes/all/GCF/002/933/455/GCF_002933455.1_ASM293345v1/GCF_002933455.1_ASM293345v1_genomic.gff.gz | ftp://ftp.ncbi.nlm.nih.gov/genomes/all/GCF/002/933/455/GCF_002933455.1_ASM293345v1/GCF_002933455.1_ASM293345v1_cds_from_genomic.fna.gz | Pathogen: clinical or host-associated sample from *Nocardia cyriacigeorgica* | Rice University | https://www.ncbi.nlm.nih.gov/data-hub/assembly/GCF_002933455.1/ |  |
| *Nocardia_cyriacigeorgica*_EML_1456 | 135487 | *Nocardia_cyri-acigeorgica* | ftp://ftp.ncbi.nlm.nih.gov/genomes/all/GCF/005/863/295/GCF_005863295.1_ASM586329v1/GCF_005863295.1_ASM586329v1_protein.faa.gz | ftp://ftp.ncbi.nlm.nih.gov/genomes/all/GCF/005/863/295/GCF_005863295.1_ASM586329v1/GCF_005863295.1_ASM586329v1_genomic.fna.gz | ftp://ftp.ncbi.nlm.nih.gov/genomes/all/GCF/005/863/295/GCF_005863295.1_ASM586329v1/GCF_005863295.1_ASM586329v1_genomic.gff.gz | ftp://ftp.ncbi.nlm.nih.gov/genomes/all/GCF/005/863/295/GCF_005863295.1_ASM586329v1/GCF_005863295.1_ASM586329v1_cds_from_genomic.fna.gz | Microbe sample from *Nocardia cyriacigeorgica* | UMR5557 Laboratoire d'Ecologie Microbienne | https://www.ncbi.nlm.nih.gov/data-hub/assembly/GCF_005863295.1/ |  |
| *Nocardia_cyriacigeorgica*_CNM20110648 | 135488 | *Nocardia_cyri-acigeorgica* | ftp://ftp.ncbi.nlm.nih.gov/genomes/all/GCF/010/868/145/GCF_010868145.1_ASM1086814v1/GCF_010868145.1_ASM1086814v1_protein.faa.gz | ftp://ftp.ncbi.nlm.nih.gov/genomes/all/GCF/010/868/145/GCF_010868145.1_ASM1086814v1/GCF_010868145.1_ASM1086814v1_genomic.fna.gz | ftp://ftp.ncbi.nlm.nih.gov/genomes/all/GCF/010/868/145/GCF_010868145.1_ASM1086814v1/GCF_010868145.1_ASM1086814v1_genomic.gff.gz | ftp://ftp.ncbi.nlm.nih.gov/genomes/all/GCF/010/868/145/GCF_010868145.1_ASM1086814v1/GCF_010868145.1_ASM1086814v1_cds_from_genomic.fna.gz | *Nocardia cyriacigeorgica* strain from soil | Instituto de Salud Carlos III | https://www.ncbi.nlm.nih.gov/data-hub/assembly/GCF_010868145.1/ |  |
| *Nocardia_cyriacigeorgica*_CNM20110624 | 135489 | *Nocardia_cyri-acigeorgica* | ftp://ftp.ncbi.nlm.nih.gov/genomes/all/GCF/010/858/045/GCF_010858045.1_ASM1085804v1/GCF_010858045.1_ASM1085804v1_protein.faa.gz | ftp://ftp.ncbi.nlm.nih.gov/genomes/all/GCF/010/858/045/GCF_010858045.1_ASM1085804v1/GCF_010858045.1_ASM1085804v1_genomic.fna.gz | ftp://ftp.ncbi.nlm.nih.gov/genomes/all/GCF/010/858/045/GCF_010858045.1_ASM1085804v1/GCF_010858045.1_ASM1085804v1_genomic.gff.gz | ftp://ftp.ncbi.nlm.nih.gov/genomes/all/GCF/010/858/045/GCF_010858045.1_ASM1085804v1/GCF_010858045.1_ASM1085804v1_cds_from_genomic.fna.gz | *Nocardia cyriacigeorgica* strain from soil | Instituto de Salud Carlos III | https://www.ncbi.nlm.nih.gov/data-hub/assembly/GCA_010858045.1/ |  |
| *Nocardia_cyriacigeorgica*_CNM20110626 | 135490 | *Nocardia_cyri-acigeorgica* | ftp://ftp.ncbi.nlm.nih.gov/genomes/all/GCF/010/858/005/GCF_010858005.1_ASM1085800v1/GCF_010858005.1_ASM1085800v1_protein.faa.gz | ftp://ftp.ncbi.nlm.nih.gov/genomes/all/GCF/010/858/005/GCF_010858005.1_ASM1085800v1/GCF_010858005.1_ASM1085800v1_genomic.fna.gz | ftp://ftp.ncbi.nlm.nih.gov/genomes/all/GCF/010/858/005/GCF_010858005.1_ASM1085800v1/GCF_010858005.1_ASM1085800v1_genomic.gff.gz | ftp://ftp.ncbi.nlm.nih.gov/genomes/all/GCF/010/858/005/GCF_010858005.1_ASM1085800v1/GCF_010858005.1_ASM1085800v1_cds_from_genomic.fna.gz | *Nocardia cyriacigeorgica* strain from soil | Instituto de Salud Carlos III | https://www.ncbi.nlm.nih.gov/data-hub/assembly/GCA_010858005.1/ |  |
| *Nocardia_cyriacigeorgica*_BJ06-0154 | 135491 | *Nocardia_cyri-acigeorgica* | ftp://ftp.ncbi.nlm.nih.gov/genomes/all/GCF/015/477/645/GCF_015477645.1_ASM1547764v1/GCF_015477645.1_ASM1547764v1_protein.faa.gz | ftp://ftp.ncbi.nlm.nih.gov/genomes/all/GCF/015/477/645/GCF_015477645.1_ASM1547764v1/GCF_015477645.1_ASM1547764v1_genomic.fna.gz | ftp://ftp.ncbi.nlm.nih.gov/genomes/all/GCF/015/477/645/GCF_015477645.1_ASM1547764v1/GCF_015477645.1_ASM1547764v1_genomic.gff.gz | ftp://ftp.ncbi.nlm.nih.gov/genomes/all/GCF/015/477/645/GCF_015477645.1_ASM1547764v1/GCF_015477645.1_ASM1547764v1_cds_from_genomic.fna.gz | Microbe sample from *Nocardia cyriacigeorgica* | Chinese PLA Center for Disease Control and Prevention | https://www.ncbi.nlm.nih.gov/data-hub/assembly/GCA_015477645.1/ |  |
| *Nocardia_cyriacigeorgica*_CNM20110649 | 135492 | *Nocardia_cyri-acigeorgica* | ftp://ftp.ncbi.nlm.nih.gov/genomes/all/GCF/010/868/115/GCF_010868115.1_ASM1086811v1/GCF_010868115.1_ASM1086811v1_protein.faa.gz | ftp://ftp.ncbi.nlm.nih.gov/genomes/all/GCF/010/868/115/GCF_010868115.1_ASM1086811v1/GCF_010868115.1_ASM1086811v1_genomic.fna.gz | ftp://ftp.ncbi.nlm.nih.gov/genomes/all/GCF/010/868/115/GCF_010868115.1_ASM1086811v1/GCF_010868115.1_ASM1086811v1_genomic.gff.gz | ftp://ftp.ncbi.nlm.nih.gov/genomes/all/GCF/010/868/115/GCF_010868115.1_ASM1086811v1/GCF_010868115.1_ASM1086811v1_cds_from_genomic.fna.gz | *Nocardia cyriacigeorgica* strain from soil | Instituto de Salud Carlos III | https://www.ncbi.nlm.nih.gov/data-hub/assembly/GCA_010868115.1/ |  |
| *Nocardia_cyriacigeorgica*_CNM20110639 | 135493 | *Nocardia_cyri-acigeorgica* | ftp://ftp.ncbi.nlm.nih.gov/genomes/all/GCF/010/868/155/GCF_010868155.1_ASM1086815v1/GCF_010868155.1_ASM1086815v1_protein.faa.gz | ftp://ftp.ncbi.nlm.nih.gov/genomes/all/GCF/010/868/155/GCF_010868155.1_ASM1086815v1/GCF_010868155.1_ASM1086815v1_genomic.fna.gz | ftp://ftp.ncbi.nlm.nih.gov/genomes/all/GCF/010/868/155/GCF_010868155.1_ASM1086815v1/GCF_010868155.1_ASM1086815v1_genomic.gff.gz | ftp://ftp.ncbi.nlm.nih.gov/genomes/all/GCF/010/868/155/GCF_010868155.1_ASM1086815v1/GCF_010868155.1_ASM1086815v1_cds_from_genomic.fna.gz | *Nocardia cyriacigeorgica* strain from soil | Instituto de Salud Carlos III | https://www.ncbi.nlm.nih.gov/data-hub/assembly/GCF_010868155.1/ |  |
| *Nocardia_cyriacigeorgica*_BJ06-0118 | 135494 | *Nocardia_cyri-acigeorgica* | ftp://ftp.ncbi.nlm.nih.gov/genomes/all/GCF/015/478/155/GCF_015478155.1_ASM1547815v1/GCF_015478155.1_ASM1547815v1_protein.faa.gz | ftp://ftp.ncbi.nlm.nih.gov/genomes/all/GCF/015/478/155/GCF_015478155.1_ASM1547815v1/GCF_015478155.1_ASM1547815v1_genomic.fna.gz | ftp://ftp.ncbi.nlm.nih.gov/genomes/all/GCF/015/478/155/GCF_015478155.1_ASM1547815v1/GCF_015478155.1_ASM1547815v1_genomic.gff.gz | ftp://ftp.ncbi.nlm.nih.gov/genomes/all/GCF/015/478/155/GCF_015478155.1_ASM1547815v1/GCF_015478155.1_ASM1547815v1_cds_from_genomic.fna.gz | Microbe sample from *Nocardia cyriacigeorgica* | Chinese PLA Center for Disease Control and Prevention | https://www.ncbi.nlm.nih.gov/data-hub/assembly/GCF_015478155.1/ |  |
| *Nocardia_amikacinitolerans* | 756689 | amikacinitolerans | <https://ftp.ncbi.nlm.nih.gov/genomes/all/GCF/001/612/615/GCF_001612615.1_ASM161261v1/GCF_001612615.1_ASM161261v1_protein.faa.gz> | https://ftp.ncbi.nlm.nih.gov/genomes/all/GCF/001/612/615/GCF_001612615.1_ASM161261v1/GCF_001612615.1_ASM161261v1_genomic.fna.gz | <https://ftp.ncbi.nlm.nih.gov/genomes/all/GCF/001/612/615/GCF_001612615.1_ASM161261v1/GCF_001612615.1_ASM161261v1_genomic.gff.gz> | <https://ftp.ncbi.nlm.nih.gov/genomes/all/GCF/001/612/615/GCF_001612615.1_ASM161261v1/GCF_001612615.1_ASM161261v1_cds_from_genomic.fna.gz> | Type strain of *Nocardia amikacinitolera-ns* | National Institute of Technology and Evaluation Biological Resource Center | https://www.ncbi.nlm.nih.gov/data-hub/assembly/GCF_001612615.1/ |  |
| *Nocardia_fusca* | 941183 | *Nocardia_fusca* | <https://ftp.ncbi.nlm.nih.gov/genomes/all/GCF/001/618/425/GCF_001618425.1_ASM161842v1/GCF_001618425.1_ASM161842v1_protein.faa.gz> | https://ftp.ncbi.nlm.nih.gov/genomes/all/GCF/001/618/425/GCF_001618425.1_ASM161842v1/GCF_001618425.1_ASM161842v1_genomic.fna.gz | https://ftp.ncbi.nlm.nih.gov/genomes/all/GCF/001/618/425/GCF_001618425.1_ASM161842v1/GCF_001618425.1_ASM161842v1_genomic.gff.gz | https://ftp.ncbi.nlm.nih.gov/genomes/all/GCF/001/618/425/GCF_001618425.1_ASM161842v1/GCF_001618425.1_ASM161842v1_cds_from_genomic.fna.gz | *Nocardia fusca* NBRC 14340 | National Institute of Technology and Evaluation Biological Resource Center | https://www.ncbi.nlm.nih.gov/data-hub/assembly/GCF_001618425.1/ |  |
| *Nocardia_mikamii* | 508464 | mikamii | <https://ftp.ncbi.nlm.nih.gov/genomes/all/GCF/001/613/505/GCF_001613505.1_ASM161350v1/GCF_001613505.1_ASM161350v1_protein.faa.gz> | https://ftp.ncbi.nlm.nih.gov/genomes/all/GCF/001/613/505/GCF_001613505.1_ASM161350v1/GCF_001613505.1_ASM161350v1_genomic.fna.gz | https://ftp.ncbi.nlm.nih.gov/genomes/all/GCF/001/613/505/GCF_001613505.1_ASM161350v1/GCF_001613505.1_ASM161350v1_genomic.gff.gz | https://ftp.ncbi.nlm.nih.gov/genomes/all/GCF/001/613/505/GCF_001613505.1_ASM161350v1/GCF_001613505.1_ASM161350v1_cds_from_genomic.fna.gz | Type strain of *Nocardia mikamii* | National Institute of Technology and Evaluation Biological Resource Center | https://www.ncbi.nlm.nih.gov/data-hub/assembly/GCF_001613505.1/ |  |
| *Nocardia_rhamnosiphila* | 426716 | rhamnosiphila | <https://ftp.ncbi.nlm.nih.gov/genomes/all/GCF/000/720/425/GCF_000720425.1_ASM72042v1/GCF_000720425.1_ASM72042v1_protein.faa.gz> | https://ftp.ncbi.nlm.nih.gov/genomes/all/GCF/000/720/425/GCF_000720425.1_ASM72042v1/GCF_000720425.1_ASM72042v1_genomic.fna.gz | https://ftp.ncbi.nlm.nih.gov/genomes/all/GCF/000/720/425/GCF_000720425.1_ASM72042v1/GCF_000720425.1_ASM72042v1_genomic.gff.gz | https://ftp.ncbi.nlm.nih.gov/genomes/all/GCF/000/720/425/GCF_000720425.1_ASM72042v1/GCF_000720425.1_ASM72042v1_cds_from_genomic.fna.gz | Microbe sample from *Nocardia rhamnosiphila* NBRC 108938 | University of Illinois | https://www.ncbi.nlm.nih.gov/data-hub/assembly/GCF_000720425.1/ |  |
| *Nocardia_niwae* | 626084 | niwae | <https://ftp.ncbi.nlm.nih.gov/genomes/all/GCF/001/613/465/GCF_001613465.1_ASM161346v1/GCF_001613465.1_ASM161346v1_protein.faa.gz> | https://ftp.ncbi.nlm.nih.gov/genomes/all/GCF/001/613/465/GCF_001613465.1_ASM161346v1/GCF_001613465.1_ASM161346v1_genomic.fna.gz | https://ftp.ncbi.nlm.nih.gov/genomes/all/GCF/001/613/465/GCF_001613465.1_ASM161346v1/GCF_001613465.1_ASM161346v1_genomic.gff.gz | https://ftp.ncbi.nlm.nih.gov/genomes/all/GCF/001/613/465/GCF_001613465.1_ASM161346v1/GCF_001613465.1_ASM161346v1_cds_from_genomic.fna.gz | Type strain of *Nocardia niwae* | National Institute of Technology and Evaluation Biological Resource Center | https://www.ncbi.nlm.nih.gov/data-hub/assembly/GCF_001613465.1/ |  |
| *Nocardia_grenadensis* | 931537 | grenadensis | <https://ftp.ncbi.nlm.nih.gov/genomes/all/GCF/001/613/445/GCF_001613445.1_ASM161344v1/GCF_001613445.1_ASM161344v1_protein.faa.gz> | https://ftp.ncbi.nlm.nih.gov/genomes/all/GCF/001/613/445/GCF_001613445.1_ASM161344v1/GCF_001613445.1_ASM161344v1_genomic.fna.gz | https://ftp.ncbi.nlm.nih.gov/genomes/all/GCF/001/613/445/GCF_001613445.1_ASM161344v1/GCF_001613445.1_ASM161344v1_genomic.gff.gz | https://ftp.ncbi.nlm.nih.gov/genomes/all/GCF/001/613/445/GCF_001613445.1_ASM161344v1/GCF_001613445.1_ASM161344v1_cds_from_genomic.fna.gz | Type strain of *Nocardia grenadensis* | National Institute of Technology and Evaluation Biological Resource Center | https://www.ncbi.nlm.nih.gov/data-hub/assembly/GCF_001613445.1/ |  |
| *Nocardia_flavorosea* | 53429 | *Nocardia_flav-orose* | <https://ftp.ncbi.nlm.nih.gov/genomes/all/GCF/001/613/385/GCF_001613385.1_ASM161338v1/GCF_001613385.1_ASM161338v1_protein.faa.gz> | <https://ftp.ncbi.nlm.nih.gov/genomes/all/GCF/001/613/385/GCF_001613385.1_ASM161338v1/GCF_001613385.1_ASM161338v1_genomic.fna.gz> | https://ftp.ncbi.nlm.nih.gov/genomes/all/GCF/001/613/385/GCF_001613385.1_ASM161338v1/GCF_001613385.1_ASM161338v1_genomic.gff.gz | https://ftp.ncbi.nlm.nih.gov/genomes/all/GCF/001/613/385/GCF_001613385.1_ASM161338v1/GCF_001613385.1_ASM161338v1_cds_from_genomic.fna.gz | Type strain of *Nocardia flavorosea* | National Institute of Technology and Evaluation Biological Resource Center | https://www.ncbi.nlm.nih.gov/data-hub/assembly/GCF_001613385.1/ |  |
| *Nocardia_yamanashiensis* | 209247 | yamanashiensis | <https://ftp.ncbi.nlm.nih.gov/genomes/all/GCF/001/613/325/GCF_001613325.1_ASM161332v1/GCF_001613325.1_ASM161332v1_protein.faa.gz> | <https://ftp.ncbi.nlm.nih.gov/genomes/all/GCF/001/613/325/GCF_001613325.1_ASM161332v1/GCF_001613325.1_ASM161332v1_genomic.fna.gz> | https://ftp.ncbi.nlm.nih.gov/genomes/all/GCF/001/613/325/GCF_001613325.1_ASM161332v1/GCF_001613325.1_ASM161332v1_genomic.gff.gz | https://ftp.ncbi.nlm.nih.gov/genomes/all/GCF/001/613/325/GCF_001613325.1_ASM161332v1/GCF_001613325.1_ASM161332v1_cds_from_genomic.fna.gz | *Nocardia yamanashiensis* NBRC 100130 | National Institute of Technology and Evaluation Biological Resource Center | https://www.ncbi.nlm.nih.gov/data-hub/assembly/GCF_001613325.1/ |  |
| *Nocardia_vermiculata* | 257274 | vermiculata | <https://ftp.ncbi.nlm.nih.gov/genomes/all/GCF/001/613/265/GCF_001613265.1_ASM161326v1/GCF_001613265.1_ASM161326v1_protein.faa.gz> | <https://ftp.ncbi.nlm.nih.gov/genomes/all/GCF/001/613/265/GCF_001613265.1_ASM161326v1/GCF_001613265.1_ASM161326v1_genomic.fna.gz> | <https://ftp.ncbi.nlm.nih.gov/genomes/all/GCF/001/613/265/GCF_001613265.1_ASM161326v1/GCF_001613265.1_ASM161326v1_genomic.gff.gz> | https://ftp.ncbi.nlm.nih.gov/genomes/all/GCF/001/613/265/GCF_001613265.1_ASM161326v1/GCF_001613265.1_ASM161326v1_cds_from_genomic.fna.gz | Type strain of *Nocardia vermiculata* | National Institute of Technology and Evaluation Biological Resource Center | https://www.ncbi.nlm.nih.gov/data-hub/assembly/GCF_001613265.1/ |  |
| *Nocardia_spelunca-e* | 419477 | *Nocardia_spel-uncae* | <https://ftp.ncbi.nlm.nih.gov/genomes/all/GCF/001/613/245/GCF_001613245.1_ASM161324v1/GCF_001613245.1_ASM161324v1_protein.faa.gz> | https://ftp.ncbi.nlm.nih.gov/genomes/all/GCF/001/613/245/GCF_001613245.1_ASM161324v1/GCF_001613245.1_ASM161324v1_genomic.fna.gz | https://ftp.ncbi.nlm.nih.gov/genomes/all/GCF/001/613/245/GCF_001613245.1_ASM161324v1/GCF_001613245.1_ASM161324v1_genomic.gff.gz | <https://ftp.ncbi.nlm.nih.gov/genomes/all/GCF/001/613/245/GCF_001613245.1_ASM161324v1/GCF_001613245.1_ASM161324v1_cds_from_genomic.fna.gz> | *Nocardia speluncae* NBRC 108251 | National Institute of Technology and Evaluation Biological Resource Center | https://www.ncbi.nlm.nih.gov/data-hub/assembly/GCF_001613245.1/ |  |
| *Nocardia_sienata* | 248552 | *Nocardia_sien-ata* | <https://ftp.ncbi.nlm.nih.gov/genomes/all/GCF/001/613/205/GCF_001613205.1_ASM161320v1/GCF_001613205.1_ASM161320v1_protein.faa.gz> | https://ftp.ncbi.nlm.nih.gov/genomes/all/GCF/001/613/205/GCF_001613205.1_ASM161320v1/GCF_001613205.1_ASM161320v1_genomic.fna.gz | https://ftp.ncbi.nlm.nih.gov/genomes/all/GCF/001/613/205/GCF_001613205.1_ASM161320v1/GCF_001613205.1_ASM161320v1_genomic.gff.gz | https://ftp.ncbi.nlm.nih.gov/genomes/all/GCF/001/613/205/GCF_001613205.1_ASM161320v1/GCF_001613205.1_ASM161320v1_cds_from_genomic.fna.gz | Type strain of *Nocardia sienata* | National Institute of Technology and Evaluation Biological Resource Center | https://www.ncbi.nlm.nih.gov/data-hub/assembly/GCF_001613205.1/ |  |
| *Nocardia_ninae* | 356145 | *Nocardia_nina-e* | <https://ftp.ncbi.nlm.nih.gov/genomes/all/GCF/007/990/755/GCF_007990755.1_ASM799075v1/GCF_007990755.1_ASM799075v1_protein.faa.gz> | https://ftp.ncbi.nlm.nih.gov/genomes/all/GCF/007/990/755/GCF_007990755.1_ASM799075v1/GCF_007990755.1_ASM799075v1_genomic.fna.gz | https://ftp.ncbi.nlm.nih.gov/genomes/all/GCF/007/990/755/GCF_007990755.1_ASM799075v1/GCF_007990755.1_ASM799075v1_genomic.gff.gz | https://ftp.ncbi.nlm.nih.gov/genomes/all/GCF/007/990/755/GCF_007990755.1_ASM799075v1/GCF_007990755.1_ASM799075v1_cds_from_genomic.fna.gz | *Nocardia ninae* NBRC 108245 | National Institute of Technology and Evaluation Biological Resource Center | https://www.ncbi.nlm.nih.gov/data-hub/assembly/GCF_007990755.1/ |  |
| *Nocardia_puris* | 208602 | puris | <https://ftp.ncbi.nlm.nih.gov/genomes/all/GCF/001/613/185/GCF_001613185.1_ASM161318v1/GCF_001613185.1_ASM161318v1_protein.faa.gz> | <https://ftp.ncbi.nlm.nih.gov/genomes/all/GCF/001/613/185/GCF_001613185.1_ASM161318v1/GCF_001613185.1_ASM161318v1_genomic.fna.gz> | <https://ftp.ncbi.nlm.nih.gov/genomes/all/GCF/001/613/185/GCF_001613185.1_ASM161318v1/GCF_001613185.1_ASM161318v1_genomic.gff.gz> | https://ftp.ncbi.nlm.nih.gov/genomes/all/GCF/001/613/185/GCF_001613185.1_ASM161318v1/GCF_001613185.1_ASM161318v1_cds_from_genomic.fna.gz | Type strain of *Nocardia puris* | National Institute of Technology and Evaluation Biological Resource Center | https://www.ncbi.nlm.nih.gov/data-hub/assembly/GCF_001613185.1/ |  |
| *Nocardia_mexican-a* | 279262 | *Nocardia_mex-icana* | <https://ftp.ncbi.nlm.nih.gov/genomes/all/GCF/001/613/165/GCF_001613165.1_ASM161316v1/GCF_001613165.1_ASM161316v1_protein.faa.gz> | <https://ftp.ncbi.nlm.nih.gov/genomes/all/GCF/001/613/165/GCF_001613165.1_ASM161316v1/GCF_001613165.1_ASM161316v1_genomic.fna.gz> | https://ftp.ncbi.nlm.nih.gov/genomes/all/GCF/001/613/165/GCF_001613165.1_ASM161316v1/GCF_001613165.1_ASM161316v1_genomic.gff.gz | https://ftp.ncbi.nlm.nih.gov/genomes/all/GCF/001/613/165/GCF_001613165.1_ASM161316v1/GCF_001613165.1_ASM161316v1_cds_from_genomic.fna.gz | *Nocardia mexicana* NBRC 108244 | National Institute of Technology and Evaluation Biological Resource Center | https://www.ncbi.nlm.nih.gov/data-hub/assembly/GCF_001613165.1/ |  |
| *Nocardia_shimofusensis* | 228596 | shimofusensis | <https://ftp.ncbi.nlm.nih.gov/genomes/all/GCF/001/613/125/GCF_001613125.1_ASM161312v1/GCF_001613125.1_ASM161312v1_protein.faa.gz> | https://ftp.ncbi.nlm.nih.gov/genomes/all/GCF/001/613/125/GCF_001613125.1_ASM161312v1/GCF_001613125.1_ASM161312v1_genomic.fna.gz | https://ftp.ncbi.nlm.nih.gov/genomes/all/GCF/001/613/125/GCF_001613125.1_ASM161312v1/GCF_001613125.1_ASM161312v1_genomic.gff.gz | https://ftp.ncbi.nlm.nih.gov/genomes/all/GCF/001/613/125/GCF_001613125.1_ASM161312v1/GCF_001613125.1_ASM161312v1_cds_from_genomic.fna.gz | Type strain of *Nocardia shimofusensis* | National Institute of Technology and Evaluation Biological Resource Center | https://www.ncbi.nlm.nih.gov/data-hub/assembly/GCF_001613125.1/ |  |
| *Nocardia_pseudobrasiliensis* | 45979 | *Nocardia_pseu-dobrasiliensis* | <https://ftp.ncbi.nlm.nih.gov/genomes/all/GCF/001/613/105/GCF_001613105.1_ASM161310v1/GCF_001613105.1_ASM161310v1_protein.faa.gz> | https://ftp.ncbi.nlm.nih.gov/genomes/all/GCF/001/613/105/GCF_001613105.1_ASM161310v1/GCF_001613105.1_ASM161310v1_genomic.fna.gz | https://ftp.ncbi.nlm.nih.gov/genomes/all/GCF/001/613/105/GCF_001613105.1_ASM161310v1/GCF_001613105.1_ASM161310v1_genomic.gff.gz | https://ftp.ncbi.nlm.nih.gov/genomes/all/GCF/001/613/105/GCF_001613105.1_ASM161310v1/GCF_001613105.1_ASM161310v1_cds_from_genomic.fna.gz | Type strain of *Nocardia pseudobrasilien-sis* | National Institute of Technology and Evaluation Biological Resource Center | https://www.ncbi.nlm.nih.gov/data-hub/assembly/GCF_001613105.1/ |  |
| *Nocardia_miyunen-sis* | 282684 | *Nocardia_miy-unensis* | <https://ftp.ncbi.nlm.nih.gov/genomes/all/GCF/001/613/065/GCF_001613065.1_ASM161306v1/GCF_001613065.1_ASM161306v1_protein.faa.gz> | https://ftp.ncbi.nlm.nih.gov/genomes/all/GCF/001/613/065/GCF_001613065.1_ASM161306v1/GCF_001613065.1_ASM161306v1_genomic.fna.gz | https://ftp.ncbi.nlm.nih.gov/genomes/all/GCF/001/613/065/GCF_001613065.1_ASM161306v1/GCF_001613065.1_ASM161306v1_genomic.gff.gz | https://ftp.ncbi.nlm.nih.gov/genomes/all/GCF/001/613/065/GCF_001613065.1_ASM161306v1/GCF_001613065.1_ASM161306v1_cds_from_genomic.fna.gz | *Nocardia miyunensis* NBRC 108239 | National Institute of Technology and Evaluation Biological Resource Center | https://www.ncbi.nlm.nih.gov/data-hub/assembly/GCF_001613065.1/ |  |
| *Nocardia_lijiangen-sis* | 299618 | *Nocardia_lijia-ngensis* | <https://ftp.ncbi.nlm.nih.gov/genomes/all/GCF/001/613/045/GCF_001613045.1_ASM161304v1/GCF_001613045.1_ASM161304v1_protein.faa.gz> | https://ftp.ncbi.nlm.nih.gov/genomes/all/GCF/001/613/045/GCF_001613045.1_ASM161304v1/GCF_001613045.1_ASM161304v1_genomic.fna.gz | https://ftp.ncbi.nlm.nih.gov/genomes/all/GCF/001/613/045/GCF_001613045.1_ASM161304v1/GCF_001613045.1_ASM161304v1_genomic.gff.gz | https://ftp.ncbi.nlm.nih.gov/genomes/all/GCF/001/613/045/GCF_001613045.1_ASM161304v1/GCF_001613045.1_ASM161304v1_cds_from_genomic.fna.gz | Type strain of *Nocardia lijiangensis* | National Institute of Technology and Evaluation Biological Resource Center | https://www.ncbi.nlm.nih.gov/data-hub/assembly/GCF_001613045.1/ |  |
| *Nocardia_jinanensi-s* | 382504 | *Nocardia_jina-nensis* | <https://ftp.ncbi.nlm.nih.gov/genomes/all/GCF/001/482/675/GCF_001482675.1_ASM148267v1/GCF_001482675.1_ASM148267v1_protein.faa.gz> | https://ftp.ncbi.nlm.nih.gov/genomes/all/GCF/001/482/675/GCF_001482675.1_ASM148267v1/GCF_001482675.1_ASM148267v1_genomic.fna.gz | <https://ftp.ncbi.nlm.nih.gov/genomes/all/GCF/001/482/675/GCF_001482675.1_ASM148267v1/GCF_001482675.1_ASM148267v1_genomic.gff.gz> | https://ftp.ncbi.nlm.nih.gov/genomes/all/GCF/001/482/675/GCF_001482675.1_ASM148267v1/GCF_001482675.1_ASM148267v1_cds_from_genomic.fna.gz | Microbe sample from *Nocardia jinanensis* NBRC 108249 | Nocardia jinanensis NBRC 108249 whole genome | https://www.ncbi.nlm.nih.gov/data-hub/assembly/GCF_001482675.1/ |  |
| *Nocardia_neocaled-oniensis* | 236511 | Neocaledonien-sis | <https://ftp.ncbi.nlm.nih.gov/genomes/all/GCF/003/182/135/GCF_003182135.1_ASM318213v1/GCF_003182135.1_ASM318213v1_protein.faa.gz> | https://ftp.ncbi.nlm.nih.gov/genomes/all/GCF/003/182/135/GCF_003182135.1_ASM318213v1/GCF_003182135.1_ASM318213v1_genomic.fna.gz | <https://ftp.ncbi.nlm.nih.gov/genomes/all/GCF/003/182/135/GCF_003182135.1_ASM318213v1/GCF_003182135.1_ASM318213v1_genomic.gff.gz> | https://ftp.ncbi.nlm.nih.gov/genomes/all/GCF/003/182/135/GCF_003182135.1_ASM318213v1/GCF_003182135.1_ASM318213v1_cds_from_genomic.fna.gz | *Nocardia neocaledoniensi-s* DSM 44717 | DOE Joint Genome Institute | https://www.ncbi.nlm.nih.gov/data-hub/assembly/GCF_003182135.1/ |  |
| *Nocardia_gamkens-is* | 352869 | gamkensis | <https://ftp.ncbi.nlm.nih.gov/genomes/all/GCF/001/612/985/GCF_001612985.1_ASM161298v1/GCF_001612985.1_ASM161298v1_protein.faa.gz> | <https://ftp.ncbi.nlm.nih.gov/genomes/all/GCF/001/612/985/GCF_001612985.1_ASM161298v1/GCF_001612985.1_ASM161298v1_genomic.fna.gz> | https://ftp.ncbi.nlm.nih.gov/genomes/all/GCF/001/612/985/GCF_001612985.1_ASM161298v1/GCF_001612985.1_ASM161298v1_genomic.gff.gz | https://ftp.ncbi.nlm.nih.gov/genomes/all/GCF/001/612/985/GCF_001612985.1_ASM161298v1/GCF_001612985.1_ASM161298v1_cds_from_genomic.fna.gz | Type strain of *Nocardia gamkensis* | National Institute of Technology and Evaluation Biological Resource Center | https://www.ncbi.nlm.nih.gov/data-hub/assembly/GCF_001612985.1/ |  |
| *Nocardia_inohanensis* | 209246 | inohanensis | <https://ftp.ncbi.nlm.nih.gov/genomes/all/GCF/001/612/945/GCF_001612945.1_ASM161294v1/GCF_001612945.1_ASM161294v1_protein.faa.gz> | https://ftp.ncbi.nlm.nih.gov/genomes/all/GCF/001/612/945/GCF_001612945.1_ASM161294v1/GCF_001612945.1_ASM161294v1_genomic.fna.gz | https://ftp.ncbi.nlm.nih.gov/genomes/all/GCF/001/612/945/GCF_001612945.1_ASM161294v1/GCF_001612945.1_ASM161294v1_genomic.gff.gz | https://ftp.ncbi.nlm.nih.gov/genomes/all/GCF/001/612/945/GCF_001612945.1_ASM161294v1/GCF_001612945.1_ASM161294v1_cds_from_genomic.fna.gz | Type strain of *Nocardia inohanensis* | National Institute of Technology and Evaluation Biological Resource Center | https://www.ncbi.nlm.nih.gov/data-hub/assembly/GCF_001612945.1/ |  |
| *Nocardia_ignorata* | 145285 | *Nocardia_igno-rata* | <https://ftp.ncbi.nlm.nih.gov/genomes/all/GCF/001/612/905/GCF_001612905.1_ASM161290v1/GCF_001612905.1_ASM161290v1_protein.faa.gz> | https://ftp.ncbi.nlm.nih.gov/genomes/all/GCF/001/612/905/GCF_001612905.1_ASM161290v1/GCF_001612905.1_ASM161290v1_genomic.fna.gz | https://ftp.ncbi.nlm.nih.gov/genomes/all/GCF/001/612/905/GCF_001612905.1_ASM161290v1/GCF_001612905.1_ASM161290v1_genomic.gff.gz | https://ftp.ncbi.nlm.nih.gov/genomes/all/GCF/001/612/905/GCF_001612905.1_ASM161290v1/GCF_001612905.1_ASM161290v1_cds_from_genomic.fna.gz | Type strain of *Nocardia ignorata* | National Institute of Technology and Evaluation Biological Resource Center | https://www.ncbi.nlm.nih.gov/data-hub/assembly/GCF_001612905.1/ |  |
| *Nocardia_harenae* | 358707 | *Nocardia_hare-nae* | https://ftp.ncbi.nlm.nih.gov/genomes/all/GCF/001/612/885/GCF_001612885.1_ASM161288v1/GCF_001612885.1_ASM161288v1_protein.faa.gz | https://ftp.ncbi.nlm.nih.gov/genomes/all/GCF/001/612/885/GCF_001612885.1_ASM161288v1/GCF_001612885.1_ASM161288v1_genomic.fna.gz | https://ftp.ncbi.nlm.nih.gov/genomes/all/GCF/001/612/885/GCF_001612885.1_ASM161288v1/GCF_001612885.1_ASM161288v1_genomic.gff.gz | https://ftp.ncbi.nlm.nih.gov/genomes/all/GCF/001/612/885/GCF_001612885.1_ASM161288v1/GCF_001612885.1_ASM161288v1_cds_from_genomic.fna.gz | Type strain of *Nocardia harenae* | National Institute of Technology and Evaluation Biological Resource Center | https://www.ncbi.nlm.nih.gov/data-hub/assembly/GCF_001612885.1/ |  |
| *Nocardia_elegans* | 300029 | elegans | https://ftp.ncbi.nlm.nih.gov/genomes/all/GCF/001/612/845/GCF_001612845.1_ASM161284v1/GCF_001612845.1_ASM161284v1_protein.faa.gz | <https://ftp.ncbi.nlm.nih.gov/genomes/all/GCF/001/612/845/GCF_001612845.1_ASM161284v1/GCF_001612845.1_ASM161284v1_genomic.fna.gz> | https://ftp.ncbi.nlm.nih.gov/genomes/all/GCF/001/612/845/GCF_001612845.1_ASM161284v1/GCF_001612845.1_ASM161284v1_genomic.gff.gz | https://ftp.ncbi.nlm.nih.gov/genomes/all/GCF/001/612/845/GCF_001612845.1_ASM161284v1/GCF_001612845.1_ASM161284v1_cds_from_genomic.fna.gz | *Nocardia elegans* NBRC 108235 | National Institute of Technology and Evaluation Biological Resource Center | https://www.ncbi.nlm.nih.gov/data-hub/assembly/GCF_001612845.1/ |  |
| *Nocardia_caishijie-nsis* | 184756 | *Nocardia_cais-hijiensis* | https://ftp.ncbi.nlm.nih.gov/genomes/all/GCF/001/612/825/GCF_001612825.1_ASM161282v1/GCF_001612825.1_ASM161282v1_protein.faa.gz | https://ftp.ncbi.nlm.nih.gov/genomes/all/GCF/001/612/825/GCF_001612825.1_ASM161282v1/GCF_001612825.1_ASM161282v1_genomic.fna.gz | <https://ftp.ncbi.nlm.nih.gov/genomes/all/GCF/001/612/825/GCF_001612825.1_ASM161282v1/GCF_001612825.1_ASM161282v1_genomic.gff.gz> | https://ftp.ncbi.nlm.nih.gov/genomes/all/GCF/001/612/825/GCF_001612825.1_ASM161282v1/GCF_001612825.1_ASM161282v1_cds_from_genomic.fna.gz | Type strain of *Nocardia caishijiensis* | National Institute of Technology and Evaluation Biological Resource Center | https://www.ncbi.nlm.nih.gov/data-hub/assembly/GCF_001612825.1/ |  |
| *Nocardia_coubleae* | 356147 | coubleae | https://ftp.ncbi.nlm.nih.gov/genomes/all/GCF/001/612/805/GCF_001612805.1_ASM161280v1/GCF_001612805.1_ASM161280v1_protein.faa.gz | <https://ftp.ncbi.nlm.nih.gov/genomes/all/GCF/001/612/805/GCF_001612805.1_ASM161280v1/GCF_001612805.1_ASM161280v1_genomic.fna.gz> | https://ftp.ncbi.nlm.nih.gov/genomes/all/GCF/001/612/805/GCF_001612805.1_ASM161280v1/GCF_001612805.1_ASM161280v1_genomic.gff.gz | https://ftp.ncbi.nlm.nih.gov/genomes/all/GCF/001/612/805/GCF_001612805.1_ASM161280v1/GCF_001612805.1_ASM161280v1_cds_from_genomic.fna.gz | *Nocardia coubleae* NBRC 108252 | National Institute of Technology and Evaluation Biological Resource Center | https://www.ncbi.nlm.nih.gov/data-hub/assembly/GCF_001612805.1/ |  |
| *Nocardia_arthritidi-s* | 228602 | *Nocardia_arth-ritidis* | https://ftp.ncbi.nlm.nih.gov/genomes/all/GCF/001/612/765/GCF_001612765.1_ASM161276v1/GCF_001612765.1_ASM161276v1_protein.faa.gz | <https://ftp.ncbi.nlm.nih.gov/genomes/all/GCF/001/612/765/GCF_001612765.1_ASM161276v1/GCF_001612765.1_ASM161276v1_genomic.fna.gz> | https://ftp.ncbi.nlm.nih.gov/genomes/all/GCF/001/612/765/GCF_001612765.1_ASM161276v1/GCF_001612765.1_ASM161276v1_genomic.gff.gz | https://ftp.ncbi.nlm.nih.gov/genomes/all/GCF/001/612/765/GCF_001612765.1_ASM161276v1/GCF_001612765.1_ASM161276v1_cds_from_genomic.fna.gz | Type strain of *Nocardia arthritidis* | National Institute of Technology and Evaluation Biological Resource Center | https://www.ncbi.nlm.nih.gov/data-hub/assembly/GCF_001612765.1/ |  |
| *Nocardia_anaemia-e* | 263910 | *Nocardia_ana-emiae* | https://ftp.ncbi.nlm.nih.gov/genomes/all/GCF/001/612/725/GCF_001612725.1_ASM161272v1/GCF_001612725.1_ASM161272v1_protein.faa.gz | <https://ftp.ncbi.nlm.nih.gov/genomes/all/GCF/001/612/725/GCF_001612725.1_ASM161272v1/GCF_001612725.1__genomic.fna.gz> | https://ftp.ncbi.nlm.nih.gov/genomes/all/GCF/001/612/725/GCF_001612725.1_ASM161272v1/GCF_001612725.1_ASM161272v1_genomic.gff.gz | <https://ftp.ncbi.nlm.nih.gov/genomes/all/GCF/001/612/725/GCF_001612725.1_ASM161272v1/GCF_001612725.1_ASM161272v1_cds_from_genomic.fna.gz> | *Nocardia anaemiae* NBRC 100462 | National Institute of Technology and Evaluation Biological Resource Center | https://www.ncbi.nlm.nih.gov/data-hub/assembly/GCF_001612725.1/ |  |
| *Nocardia_altamire-nsis* | 472158 | altamirensis | https://ftp.ncbi.nlm.nih.gov/genomes/all/GCF/001/612/685/GCF_001612685.1_ASM161268v1/GCF_001612685.1_ASM161268v1_protein.faa.gz | https://ftp.ncbi.nlm.nih.gov/genomes/all/GCF/001/612/685/GCF_001612685.1_ASM161268v1/GCF_001612685.1_ASM161268v1_genomic.fna.gz | https://ftp.ncbi.nlm.nih.gov/genomes/all/GCF/001/612/685/GCF_001612685.1_ASM161268v1/GCF_001612685.1_ASM161268v1_genomic.gff.gz | https://ftp.ncbi.nlm.nih.gov/genomes/all/GCF/001/612/685/GCF_001612685.1_ASM161268v1/GCF_001612685.1_ASM161268v1_cds_from_genomic.fna.gz | *Nocardia altamirensis* NBRC 108246 | National Institute of Technology and Evaluation Biological Resource Center | https://www.ncbi.nlm.nih.gov/data-hub/assembly/GCF_001612685.1/ |  |
| *Nocardia_alba* | 225051 | *Nocardia_alba* | https://ftp.ncbi.nlm.nih.gov/genomes/all/GCF/001/612/665/GCF_001612665.1_ASM161266v1/GCF_001612665.1_ASM161266v1_protein.faa.gz | https://ftp.ncbi.nlm.nih.gov/genomes/all/GCF/001/612/665/GCF_001612665.1_ASM161266v1/GCF_001612665.1_ASM161266v1_genomic.fna.gz | https://ftp.ncbi.nlm.nih.gov/genomes/all/GCF/001/612/665/GCF_001612665.1_ASM161266v1/GCF_001612665.1_ASM161266v1_genomic.gff.gz | <https://ftp.ncbi.nlm.nih.gov/genomes/all/GCF/001/612/665/GCF_001612665.1_ASM161266v1/GCF_001612665.1_ASM161266v1_cds_from_genomic.fna.gz> | *Nocardia alba* NBRC 108234 | National Institute of Technology and Evaluation Biological Resource Center | https://www.ncbi.nlm.nih.gov/data-hub/assembly/GCF_001612665.1/ |  |
| *Nocardia_acidivor-ans* | 404580 | acidivorans | https://ftp.ncbi.nlm.nih.gov/genomes/all/GCF/001/625/085/GCF_001625085.1_ASM162508v1/GCF_001625085.1_ASM162508v1_protein.faa.gz | https://ftp.ncbi.nlm.nih.gov/genomes/all/GCF/001/625/085/GCF_001625085.1_ASM162508v1/GCF_001625085.1_ASM162508v1_genomic.fna.gz | https://ftp.ncbi.nlm.nih.gov/genomes/all/GCF/001/625/085/GCF_001625085.1_ASM162508v1/GCF_001625085.1_ASM162508v1_genomic.gff.gz | https://ftp.ncbi.nlm.nih.gov/genomes/all/GCF/001/625/085/GCF_001625085.1_ASM162508v1/GCF_001625085.1_ASM162508v1_cds_from_genomic.fna.gz | Type strain of *Nocardia acidivorans* | National Institute of Technology and Evaluation Biological Resource Center | https://www.ncbi.nlm.nih.gov/data-hub/assembly/GCF_001625085.1/ |  |
| *Nocardia_xishanen-sis* | 238964 | *Nocardia_xish-anensis* | https://ftp.ncbi.nlm.nih.gov/genomes/all/GCF/001/613/365/GCF_001613365.1_ASM161336v1/GCF_001613365.1_ASM161336v1_protein.faa.gz | https://ftp.ncbi.nlm.nih.gov/genomes/all/GCF/001/613/365/GCF_001613365.1_ASM161336v1/GCF_001613365.1_ASM161336v1_genomic.fna.gz | https://ftp.ncbi.nlm.nih.gov/genomes/all/GCF/001/613/365/GCF_001613365.1_ASM161336v1/GCF_001613365.1_ASM161336v1_genomic.gff.gz | https://ftp.ncbi.nlm.nih.gov/genomes/all/GCF/001/613/365/GCF_001613365.1_ASM161336v1/GCF_001613365.1_ASM161336v1_cds_from_genomic.fna.gz | Type strain of *Nocardia xishanensis* | National Institute of Technology and Evaluation Biological Resource Center | https://www.ncbi.nlm.nih.gov/data-hub/assembly/GCF_001613365.1/ |  |
| *Nocardia_uniformi-s* | 53432 | *Nocardia_unif-ormis* | https://ftp.ncbi.nlm.nih.gov/genomes/all/GCF/001/613/345/GCF_001613345.1_ASM161334v1/GCF_001613345.1_ASM161334v1_protein.faa.gz | <https://ftp.ncbi.nlm.nih.gov/genomes/all/GCF/001/613/345/GCF_001613345.1_ASM161334v1/GCF_001613345.1_ASM161334v1_genomic.fna.gz> | https://ftp.ncbi.nlm.nih.gov/genomes/all/GCF/001/613/345/GCF_001613345.1_ASM161334v1/GCF_001613345.1_ASM161334v1_genomic.gff.gz | https://ftp.ncbi.nlm.nih.gov/genomes/all/GCF/001/613/345/GCF_001613345.1_ASM161334v1/GCF_001613345.1_ASM161334v1_cds_from_genomic.fna.gz | Type strain of *Nocardia uniformis* | National Institute of Technology and Evaluation Biological Resource Center | https://www.ncbi.nlm.nih.gov/data-hub/assembly/GCF_001613345.1/ |  |
| *Nocardia_vaccinii* | 1822 | vaccinii | https://ftp.ncbi.nlm.nih.gov/genomes/all/GCF/001/613/305/GCF_001613305.1_ASM161330v1/GCF_001613305.1_ASM161330v1_protein.faa.gz | https://ftp.ncbi.nlm.nih.gov/genomes/all/GCF/001/613/305/GCF_001613305.1_ASM161330v1/GCF_001613305.1_ASM161330v1_genomic.fna.gz | https://ftp.ncbi.nlm.nih.gov/genomes/all/GCF/001/613/305/GCF_001613305.1_ASM161330v1/GCF_001613305.1_ASM161330v1_genomic.gff.gz | https://ftp.ncbi.nlm.nih.gov/genomes/all/GCF/001/613/305/GCF_001613305.1_ASM161330v1/GCF_001613305.1_ASM161330v1_cds_from_genomic.fna.gz | Type strain of *Nocardia vaccinii* | National Institute of Technology and Evaluation Biological Resource Center | https://www.ncbi.nlm.nih.gov/data-hub/assembly/GCF_001613305.1/ |  |
| *Nocardia_seriolae* | 37332 | seriolae | https://ftp.ncbi.nlm.nih.gov/genomes/all/GCF/001/865/855/GCF_001865855.1_ASM186585v1/GCF_001865855.1_ASM186585v1_protein.faa.gz | <https://ftp.ncbi.nlm.nih.gov/genomes/all/GCF/001/865/855/GCF_001865855.1_ASM186585v1/GCF_001865855.1_ASM186585v1_genomic.fna.gz> | https://ftp.ncbi.nlm.nih.gov/genomes/all/GCF/001/865/855/GCF_001865855.1_ASM186585v1/GCF_001865855.1_ASM186585v1_genomic.gff.gz | https://ftp.ncbi.nlm.nih.gov/genomes/all/GCF/001/865/855/GCF_001865855.1_ASM186585v1/GCF_001865855.1_ASM186585v1_cds_from_genomic.fna.gz | Genome sequencing of *Nocardia seriolae* strain EM150506 | National Institute of Fisheries Science | https://www.ncbi.nlm.nih.gov/data-hub/assembly/GCF_001865855.1/ |  |
| *Nocardia_pseudov-accinii* | 189540 | pseudovaccinii | https://ftp.ncbi.nlm.nih.gov/genomes/all/GCF/001/613/225/GCF_001613225.1_ASM161322v1/GCF_001613225.1_ASM161322v1_protein.faa.gz | https://ftp.ncbi.nlm.nih.gov/genomes/all/GCF/001/613/225/GCF_001613225.1_ASM161322v1/GCF_001613225.1_ASM161322v1_genomic.fna.gz | https://ftp.ncbi.nlm.nih.gov/genomes/all/GCF/001/613/225/GCF_001613225.1_ASM161322v1/GCF_001613225.1_ASM161322v1_genomic.gff.gz | https://ftp.ncbi.nlm.nih.gov/genomes/all/GCF/001/613/225/GCF_001613225.1_ASM161322v1/GCF_001613225.1_ASM161322v1_cds_from_genomic.fna.gz | *Nocardia pseudovaccinii* NBRC 100343 | National Institute of Technology and Evaluation Biological Resource Center | https://www.ncbi.nlm.nih.gov/data-hub/assembly/GCF_001613225.1/ |  |
| *Nocardia_jejuensis* | 328049 | jejuensis | https://ftp.ncbi.nlm.nih.gov/genomes/all/GCF/001/613/145/GCF_001613145.1_ASM161314v1/GCF_001613145.1_ASM161314v1_protein.faa.gz | https://ftp.ncbi.nlm.nih.gov/genomes/all/GCF/001/613/145/GCF_001613145.1_ASM161314v1/GCF_001613145.1_ASM161314v1_genomic.fna.gz | https://ftp.ncbi.nlm.nih.gov/genomes/all/GCF/001/613/145/GCF_001613145.1_ASM161314v1/GCF_001613145.1_ASM161314v1_genomic.gff.gz | https://ftp.ncbi.nlm.nih.gov/genomes/all/GCF/001/613/145/GCF_001613145.1_ASM161314v1/GCF_001613145.1_ASM161314v1_cds_from_genomic.fna.gz | *Nocardia jejuensis* NBRC 103114 | National Institute of Technology and Evaluation Biological Resource Center | https://www.ncbi.nlm.nih.gov/data-hub/assembly/GCF_001613145.1/ |  |
| *Nocardia_kruczaki-ae* | 261477 | kruczakiae | https://ftp.ncbi.nlm.nih.gov/genomes/all/GCF/001/612/965/GCF_001612965.1_ASM161296v1/GCF_001612965.1_ASM161296v1_protein.faa.gz | https://ftp.ncbi.nlm.nih.gov/genomes/all/GCF/001/612/965/GCF_001612965.1_ASM161296v1/GCF_001612965.1_ASM161296v1_genomic.fna.gz | https://ftp.ncbi.nlm.nih.gov/genomes/all/GCF/001/612/965/GCF_001612965.1_ASM161296v1/GCF_001612965.1_ASM161296v1_genomic.gff.gz | https://ftp.ncbi.nlm.nih.gov/genomes/all/GCF/001/612/965/GCF_001612965.1_ASM161296v1/GCF_001612965.1_ASM161296v1_cds_from_genomic.fna.gz | Type strain of *Nocardia kruczakiae* | National Institute of Technology and Evaluation Biological Resource Center | https://www.ncbi.nlm.nih.gov/data-hub/assembly/GCF_001612965.1/ |  |
| *Nocardia_beijingen-sis* | 95162 | *Nocardia_beiji-ngensis* | https://ftp.ncbi.nlm.nih.gov/genomes/all/GCF/001/612/785/GCF_001612785.1_ASM161278v1/GCF_001612785.1_ASM161278v1_protein.faa.gz | <https://ftp.ncbi.nlm.nih.gov/genomes/all/GCF/001/612/785/GCF_001612785.1_ASM161278v1/GCF_001612785.1_ASM161278v1_genomic.fna.gz> | https://ftp.ncbi.nlm.nih.gov/genomes/all/GCF/001/612/785/GCF_001612785.1_ASM161278v1/GCF_001612785.1_ASM161278v1_genomic.gff.gz | https://ftp.ncbi.nlm.nih.gov/genomes/all/GCF/001/612/785/GCF_001612785.1_ASM161278v1/GCF_001612785.1_ASM161278v1_cds_from_genomic.fna.gz | Type strain of *Nocardia beijingensis* | National Institute of Technology and Evaluation Biological Resource Center | https://www.ncbi.nlm.nih.gov/data-hub/assembly/GCF_001612785.1/ |  |
| *Nocardia_amamien-sis* | 404578 | amamiensis | https://ftp.ncbi.nlm.nih.gov/genomes/all/GCF/001/612/745/GCF_001612745.1_ASM161274v1/GCF_001612745.1_ASM161274v1_protein.faa.gz | <https://ftp.ncbi.nlm.nih.gov/genomes/all/GCF/001/612/745/GCF_001612745.1_ASM161274v1/GCF_001612745.1_ASM161274v1_genomic.fna.gz> | https://ftp.ncbi.nlm.nih.gov/genomes/all/GCF/001/612/745/GCF_001612745.1_ASM161274v1/GCF_001612745.1_ASM161274v1_genomic.gff.gz | https://ftp.ncbi.nlm.nih.gov/genomes/all/GCF/001/612/745/GCF_001612745.1_ASM161274v1/GCF_001612745.1_ASM161274v1_cds_from_genomic.fna.gz | *Nocardia amamiensis* NBRC 102102 | National Institute of Technology and Evaluation Biological Resource Center | https://www.ncbi.nlm.nih.gov/data-hub/assembly/GCF_001612745.1/ |  |
| *Nocardia_africana* | 134964 | *Nocardia_afri-cana* | https://ftp.ncbi.nlm.nih.gov/genomes/all/GCF/001/612/635/GCF_001612635.1_ASM161263v1/GCF_001612635.1_ASM161263v1_protein.faa.gz | <https://ftp.ncbi.nlm.nih.gov/genomes/all/GCF/001/612/635/GCF_001612635.1_ASM161263v1/GCF_001612635.1_ASM161263v1_genomic.fna.gz> | https://ftp.ncbi.nlm.nih.gov/genomes/all/GCF/001/612/635/GCF_001612635.1_ASM161263v1/GCF_001612635.1_ASM161263v1_genomic.gff.gz | https://ftp.ncbi.nlm.nih.gov/genomes/all/GCF/001/612/635/GCF_001612635.1_ASM161263v1/GCF_001612635.1_ASM161263v1_cds_from_genomic.fna.gz | *Nocardia africana* NBRC 100379 | National Institute of Technology and Evaluation Biological Resource Center | https://www.ncbi.nlm.nih.gov/data-hub/assembly/GCF_001612635.1/ |  |
| *Nocardia_transvalensis* | 37333 | *Nocardia_tran-svalensis* | <https://ftp.ncbi.nlm.nih.gov/genomes/all/GCF/000/308/875/GCF_000308875.1_ASM30887v1/GCF_000308875.1_ASM30887v1_protein.faa.gz> | https://ftp.ncbi.nlm.nih.gov/genomes/all/GCF/000/308/875/GCF_000308875.1_ASM30887v1/GCF_000308875.1_ASM30887v1_genomic.fna.gz | https://ftp.ncbi.nlm.nih.gov/genomes/all/GCF/000/308/875/GCF_000308875.1_ASM30887v1/GCF_000308875.1_ASM30887v1_genomic.gff.gz | https://ftp.ncbi.nlm.nih.gov/genomes/all/GCF/000/308/875/GCF_000308875.1_ASM30887v1/GCF_000308875.1_ASM30887v1_cds_from_genomic.fna.gz | Generic sample from *Nocardia transvalensis* NBRC 15921 | National Institute of Technology and Evaluation Biological Resource Center | https://www.ncbi.nlm.nih.gov/data-hub/assembly/GCF_000308875.1/ |  |
| *Nocardia_veterana* | 132249 | veterana | https://ftp.ncbi.nlm.nih.gov/genomes/all/GCF/000/308/855/GCF_000308855.1_ASM30885v1/GCF_000308855.1_ASM30885v1_protein.faa.gz | <ftp://ftp.ncbi.nlm.nih.gov/genomes/all/GCF/000/308/855/GCF_000308855.1_ASM30885v1/GCF_000308855.1_ASM30885v1_genomic.fna.gz> | https://ftp.ncbi.nlm.nih.gov/genomes/all/GCF/000/308/855/GCF_000308855.1_ASM30885v1/GCF_000308855.1_ASM30885v1_genomic.gff.gz | https://ftp.ncbi.nlm.nih.gov/genomes/all/GCF/000/308/855/GCF_000308855.1_ASM30885v1/GCF_000308855.1_ASM30885v1_cds_from_genomic.fna.gz | Generic sample from *Nocardia veterana* NBRC 100344 | National Institute of Technology and Evaluation Biological Resource Center | https://www.ncbi.nlm.nih.gov/data-hub/assembly/GCF_000308855.1/ |  |
| *Nocardia_vinacea* | 96468 | vinacea | https://ftp.ncbi.nlm.nih.gov/genomes/all/GCF/000/308/835/GCF_000308835.1_ASM30883v1/GCF_000308835.1_ASM30883v1_protein.faa.gz | https://ftp.ncbi.nlm.nih.gov/genomes/all/GCF/000/308/835/GCF_000308835.1_ASM30883v1/GCF_000308835.1_ASM30883v1_genomic.fna.gz | https://ftp.ncbi.nlm.nih.gov/genomes/all/GCF/000/308/835/GCF_000308835.1_ASM30883v1/GCF_000308835.1_ASM30883v1_genomic.gff.gz | https://ftp.ncbi.nlm.nih.gov/genomes/all/GCF/000/308/835/GCF_000308835.1_ASM30883v1/GCF_000308835.1_ASM30883v1_cds_from_genomic.fna.gz | Generic sample from *Nocardia vinacea* NBRC 16497 | National Institute of Technology and Evaluation Biological Resource Center | https://www.ncbi.nlm.nih.gov/data-hub/assembly/GCF_000308835.1/ |  |
| *Nocardia_concava* | 257281 | concava | https://ftp.ncbi.nlm.nih.gov/genomes/all/GCF/000/308/815/GCF_000308815.1_ASM30881v1/GCF_000308815.1_ASM30881v1_protein.faa.gz | https://ftp.ncbi.nlm.nih.gov/genomes/all/GCF/000/308/815/GCF_000308815.1_ASM30881v1/GCF_000308815.1_ASM30881v1_genomic.fna.gz | https://ftp.ncbi.nlm.nih.gov/genomes/all/GCF/000/308/815/GCF_000308815.1_ASM30881v1/GCF_000308815.1_ASM30881v1_genomic.gff.gz | https://ftp.ncbi.nlm.nih.gov/genomes/all/GCF/000/308/815/GCF_000308815.1_ASM30881v1/GCF_000308815.1_ASM30881v1_cds_from_genomic.fna.gz | Generic sample from *Nocardia concava* NBRC 100430 | National Institute of Technology and Evaluation Biological Resource Center | https://www.ncbi.nlm.nih.gov/data-hub/assembly/GCF_000308815.1/ |  |
| *Nocardia_thailandi-ca* | 257275 | *Nocardia_thail-andica* | https://ftp.ncbi.nlm.nih.gov/genomes/all/GCF/000/308/795/GCF_000308795.1_ASM30879v1/GCF_000308795.1_ASM30879v1_protein.faa.gz | https://ftp.ncbi.nlm.nih.gov/genomes/all/GCF/000/308/795/GCF_000308795.1_ASM30879v1/GCF_000308795.1_ASM30879v1_genomic.fna.gz | https://ftp.ncbi.nlm.nih.gov/genomes/all/GCF/000/308/795/GCF_000308795.1_ASM30879v1/GCF_000308795.1_ASM30879v1_genomic.gff.gz | https://ftp.ncbi.nlm.nih.gov/genomes/all/GCF/000/308/795/GCF_000308795.1_ASM30879v1/GCF_000308795.1_ASM30879v1_cds_from_genomic.fna.gz | Generic sample from *Nocardia thailandica* NBRC 100428 | National Institute of Technology and Evaluation Biological Resource Center | https://www.ncbi.nlm.nih.gov/data-hub/assembly/GCF_000308795.1/ |  |
| *Nocardia_testacea* | 248551 | testacea | https://ftp.ncbi.nlm.nih.gov/genomes/all/GCF/000/308/775/GCF_000308775.1_ASM30877v1/GCF_000308775.1_ASM30877v1_protein.faa.gz | https://ftp.ncbi.nlm.nih.gov/genomes/all/GCF/000/308/775/GCF_000308775.1_ASM30877v1/GCF_000308775.1_ASM30877v1_genomic.fna.gz | https://ftp.ncbi.nlm.nih.gov/genomes/all/GCF/000/308/775/GCF_000308775.1_ASM30877v1/GCF_000308775.1_ASM30877v1_genomic.gff.gz | https://ftp.ncbi.nlm.nih.gov/genomes/all/GCF/000/308/775/GCF_000308775.1_ASM30877v1/GCF_000308775.1_ASM30877v1_cds_from_genomic.fna.gz | Generic sample from *Nocardia testacea* NBRC 100365 | National Institute of Technology and Evaluation Biological Resource Center | https://www.ncbi.nlm.nih.gov/data-hub/assembly/GCF_000308775.1/ |  |
| *Nocardia_terpenic-a* | 455432 | terpenica | https://ftp.ncbi.nlm.nih.gov/genomes/all/GCF/001/625/105/GCF_001625105.1_ASM162510v1/GCF_001625105.1_ASM162510v1_protein.faa.gz | <https://ftp.ncbi.nlm.nih.gov/genomes/all/GCF/001/625/105/GCF_001625105.1_ASM162510v1/GCF_001625105.1_ASM162510v1_genomic.fna.gz> | https://ftp.ncbi.nlm.nih.gov/genomes/all/GCF/001/625/105/GCF_001625105.1_ASM162510v1/GCF_001625105.1_ASM162510v1_genomic.gff.gz | https://ftp.ncbi.nlm.nih.gov/genomes/all/GCF/001/625/105/GCF_001625105.1_ASM162510v1/GCF_001625105.1_ASM162510v1_cds_from_genomic.fna.gz | *Nocardia terpenica* IFM 0406 clinical isolate from a patient of lung nocardiosis | University of Tuebingen | https://www.ncbi.nlm.nih.gov/data-hub/assembly/GCF_001625105.1/ |  |
| *Nocardia_pneumoniae* | 228601 | pneumoniae | https://ftp.ncbi.nlm.nih.gov/genomes/all/GCF/000/308/755/GCF_000308755.1_ASM30875v1/GCF_000308755.1_ASM30875v1_protein.faa.gz | https://ftp.ncbi.nlm.nih.gov/genomes/all/GCF/000/308/755/GCF_000308755.1_ASM30875v1/GCF_000308755.1_ASM30875v1_genomic.fna.gz | https://ftp.ncbi.nlm.nih.gov/genomes/all/GCF/000/308/755/GCF_000308755.1_ASM30875v1/GCF_000308755.1_ASM30875v1_genomic.gff.gz | https://ftp.ncbi.nlm.nih.gov/genomes/all/GCF/000/308/755/GCF_000308755.1_ASM30875v1/GCF_000308755.1_ASM30875v1_cds_from_genomic.fna.gz | Generic sample from *Nocardia pneumoniae* NBRC 100136 | National Institute of Technology and Evaluation Biological Resource Center | https://www.ncbi.nlm.nih.gov/data-hub/assembly/GCF_000308755.1/ |  |
| *Nocardia_tenerifen-sis* | 228006 | *Nocardia_tene-rifensis* | https://ftp.ncbi.nlm.nih.gov/genomes/all/GCF/000/308/715/GCF_000308715.1_ASM30871v1/GCF_000308715.1_ASM30871v1_protein.faa.gz | <https://ftp.ncbi.nlm.nih.gov/genomes/all/GCF/000/308/715/GCF_000308715.1_ASM30871v1/GCF_000308715.1_ASM30871v1_genomic.fna.gz> | https://ftp.ncbi.nlm.nih.gov/genomes/all/GCF/000/308/715/GCF_000308715.1_ASM30871v1/GCF_000308715.1_ASM30871v1_genomic.gff.gz | https://ftp.ncbi.nlm.nih.gov/genomes/all/GCF/000/308/715/GCF_000308715.1_ASM30871v1/GCF_000308715.1_ASM30871v1_cds_from_genomic.fna.gz | Generic sample from *Nocardia tenerifensis* NBRC 101015 | National Institute of Technology and Evaluation Biological Resource Center | https://www.ncbi.nlm.nih.gov/data-hub/assembly/GCF_000308715.1/ |  |
| *Nocardia_takedens-is* | 259390 | *Nocardia_take-densis* | https://ftp.ncbi.nlm.nih.gov/genomes/all/GCF/000/308/695/GCF_000308695.1_ASM30869v1/GCF_000308695.1_ASM30869v1_protein.faa.gz | https://ftp.ncbi.nlm.nih.gov/genomes/all/GCF/000/308/695/GCF_000308695.1_ASM30869v1/GCF_000308695.1_ASM30869v1_genomic.fna.gz | https://ftp.ncbi.nlm.nih.gov/genomes/all/GCF/000/308/695/GCF_000308695.1_ASM30869v1/GCF_000308695.1_ASM30869v1_genomic.gff.gz | https://ftp.ncbi.nlm.nih.gov/genomes/all/GCF/000/308/695/GCF_000308695.1_ASM30869v1/GCF_000308695.1_ASM30869v1_cds_from_genomic.fna.gz | Generic sample from *Nocardia takedensis* NBRC 100417 | National Institute of Technology and Evaluation Biological Resource Center |  |  |
| *Nocardia_paucivor-ans* | 114259 | *Nocardia_pau-civorans* | https://ftp.ncbi.nlm.nih.gov/genomes/all/GCF/000/308/675/GCF_000308675.1_ASM30867v1/GCF_000308675.1_ASM30867v1_protein.faa.gz | https://ftp.ncbi.nlm.nih.gov/genomes/all/GCF/000/308/675/GCF_000308675.1_ASM30867v1/GCF_000308675.1_ASM30867v1_genomic.fna.gz | https://ftp.ncbi.nlm.nih.gov/genomes/all/GCF/000/308/675/GCF_000308675.1_ASM30867v1/GCF_000308675.1_ASM30867v1_genomic.gff.gz | https://ftp.ncbi.nlm.nih.gov/genomes/all/GCF/000/308/675/GCF_000308675.1_ASM30867v1/GCF_000308675.1_ASM30867v1_cds_from_genomic.fna.gz | Generic sample from *Nocardia paucivorans* NBRC 100373 | National Institute of Technology and Evaluation Biological Resource Center | https://www.ncbi.nlm.nih.gov/data-hub/assembly/GCF_000308675.1/ |  |
| *Nocardia_niigatensis* | 209249 | niigatensis | https://ftp.ncbi.nlm.nih.gov/genomes/all/GCF/000/308/655/GCF_000308655.1_ASM30865v1/GCF_000308655.1_ASM30865v1_protein.faa.gz | https://ftp.ncbi.nlm.nih.gov/genomes/all/GCF/000/308/655/GCF_000308655.1_ASM30865v1/GCF_000308655.1_ASM30865v1_genomic.fna.gz | https://ftp.ncbi.nlm.nih.gov/genomes/all/GCF/000/308/655/GCF_000308655.1_ASM30865v1/GCF_000308655.1_ASM30865v1_genomic.gff.gz | https://ftp.ncbi.nlm.nih.gov/genomes/all/GCF/000/308/655/GCF_000308655.1_ASM30865v1/GCF_000308655.1_ASM30865v1_cds_from_genomic.fna.gz | Generic sample from *Nocardia niigatensis* NBRC 100131 | National Institute of Technology and Evaluation Biological Resource Center | https://www.ncbi.nlm.nih.gov/data-hub/assembly/GCF_000308655.1/ |  |
| *Nocardia_jiangxien-sis* | 282685 | *Nocardia_jian-gxiensis* | https://ftp.ncbi.nlm.nih.gov/genomes/all/GCF/000/308/615/GCF_000308615.1_ASM30861v1/GCF_000308615.1_ASM30861v1_protein.faa.gz | https://ftp.ncbi.nlm.nih.gov/genomes/all/GCF/000/308/615/GCF_000308615.1_ASM30861v1/GCF_000308615.1_ASM30861v1_genomic.fna.gz | https://ftp.ncbi.nlm.nih.gov/genomes/all/GCF/000/308/615/GCF_000308615.1_ASM30861v1/GCF_000308615.1_ASM30861v1_genomic.gff.gz | https://ftp.ncbi.nlm.nih.gov/genomes/all/GCF/000/308/615/GCF_000308615.1_ASM30861v1/GCF_000308615.1_ASM30861v1_cds_from_genomic.fna.gz | Generic sample from *Nocardia jiangxiensis* NBRC 101359 | National Institute of Technology and Evaluation Biological Resource Center |  |  |
| *Nocardia_higoensi-s* | 228599 | higoensis | https://ftp.ncbi.nlm.nih.gov/genomes/all/GCF/000/308/595/GCF_000308595.1_ASM30859v1/GCF_000308595.1_ASM30859v1_protein.faa.gz | <https://ftp.ncbi.nlm.nih.gov/genomes/all/GCF/000/308/595/GCF_000308595.1_ASM30859v1/GCF_000308595.1_ASM30859v1_genomic.fna.gz> | https://ftp.ncbi.nlm.nih.gov/genomes/all/GCF/000/308/595/GCF_000308595.1_ASM30859v1/GCF_000308595.1_ASM30859v1_genomic.gff.gz | https://ftp.ncbi.nlm.nih.gov/genomes/all/GCF/000/308/595/GCF_000308595.1_ASM30859v1/GCF_000308595.1_ASM30859v1_cds_from_genomic.fna.gz | Generic sample from *Nocardia higoensis* NBRC 100133 | National Institute of Technology and Evaluation Biological Resource Center | https://www.ncbi.nlm.nih.gov/data-hub/assembly/GCF_000308595.1/ |  |
| *Nocardia_exalbida* | 290231 | *Nocardia_exal-bida* | https://ftp.ncbi.nlm.nih.gov/genomes/all/GCF/000/308/575/GCF_000308575.1_ASM30857v1/GCF_000308575.1_ASM30857v1_protein.faa.gz | https://ftp.ncbi.nlm.nih.gov/genomes/all/GCF/000/308/575/GCF_000308575.1_ASM30857v1/GCF_000308575.1_ASM30857v1_genomic.fna.gz | https://ftp.ncbi.nlm.nih.gov/genomes/all/GCF/000/308/575/GCF_000308575.1_ASM30857v1/GCF_000308575.1_ASM30857v1_genomic.gff.gz | https://ftp.ncbi.nlm.nih.gov/genomes/all/GCF/000/308/575/GCF_000308575.1_ASM30857v1/GCF_000308575.1_ASM30857v1_cds_from_genomic.fna.gz | Generic sample from *Nocardia exalbida* NBRC 100660 | National Institute of Technology and Evaluation Biological Resource Center | https://www.ncbi.nlm.nih.gov/data-hub/assembly/GCF_000308575.1/ |  |
| *Nocardia_cerradoe-nsis* |  | cerradoensis | https://ftp.ncbi.nlm.nih.gov/genomes/all/GCF/000/308/535/GCF_000308535.1_ASM30853v1/GCF_000308535.1_ASM30853v1_protein.faa.gz | https://ftp.ncbi.nlm.nih.gov/genomes/all/GCF/000/308/535/GCF_000308535.1_ASM30853v1/GCF_000308535.1_ASM30853v1_genomic.fna.gz | https://ftp.ncbi.nlm.nih.gov/genomes/all/GCF/000/308/535/GCF_000308535.1_ASM30853v1/GCF_000308535.1_ASM30853v1_genomic.gff.gz | <https://ftp.ncbi.nlm.nih.gov/genomes/all/GCF/000/308/535/GCF_000308535.1_ASM30853v1/GCF_000308535.1_ASM30853v1_cds_from_genomic.fna.gz> | Generic sample from *Nocardia cerradoensis* NBRC 101014 | National Institute of Technology and Evaluation Biological Resource Center | https://www.ncbi.nlm.nih.gov/data-hub/assembly/GCF_000308535.1/ |  |
| *Nocardia_carnea* | 37328 | *Nocardia_carn-ea* | https://ftp.ncbi.nlm.nih.gov/genomes/all/GCF/000/308/515/GCF_000308515.1_ASM30851v1/GCF_000308515.1_ASM30851v1_protein.faa.gz | https://ftp.ncbi.nlm.nih.gov/genomes/all/GCF/000/308/515/GCF_000308515.1_ASM30851v1/GCF_000308515.1_ASM30851v1_genomic.fna.gz | <https://ftp.ncbi.nlm.nih.gov/genomes/all/GCF/000/308/515/GCF_000308515.1_ASM30851v1/GCF_000308515.1_ASM30851v1_genomic.gff.gz> | https://ftp.ncbi.nlm.nih.gov/genomes/all/GCF/000/308/515/GCF_000308515.1_ASM30851v1/GCF_000308515.1_ASM30851v1_cds_from_genomic.fna.gz | Generic sample from *Nocardia carnea* NBRC 14403 | National Institute of Technology and Evaluation Biological Resource Center | https://www.ncbi.nlm.nih.gov/data-hub/assembly/GCF_000308515.1/ |  |
| *Nocardia_brevicate-na* | 37327 | *Nocardia_brev-icatena* | https://ftp.ncbi.nlm.nih.gov/genomes/all/GCF/000/308/495/GCF_000308495.1_ASM30849v1/GCF_000308495.1_ASM30849v1_protein.faa.gz | https://ftp.ncbi.nlm.nih.gov/genomes/all/GCF/000/308/495/GCF_000308495.1_ASM30849v1/GCF_000308495.1_ASM30849v1_genomic.fna.gz | https://ftp.ncbi.nlm.nih.gov/genomes/all/GCF/000/308/495/GCF_000308495.1_ASM30849v1/GCF_000308495.1_ASM30849v1_genomic.gff.gz | https://ftp.ncbi.nlm.nih.gov/genomes/all/GCF/000/308/495/GCF_000308495.1_ASM30849v1/GCF_000308495.1_ASM30849v1_cds_from_genomic.fna.gz | Generic sample from *Nocardia brevicatena* NBRC 12119 | National Institute of Technology and Evaluation Biological Resource Center | https://www.ncbi.nlm.nih.gov/data-hub/assembly/GCF_000308495.1/ |  |
| *Nocardia_abscessu-s* | 120957 | *Nocardia_absc-essus* | https://ftp.ncbi.nlm.nih.gov/genomes/all/GCF/000/308/455/GCF_000308455.1_ASM30845v1/GCF_000308455.1_ASM30845v1_protein.faa.gz | https://ftp.ncbi.nlm.nih.gov/genomes/all/GCF/000/308/455/GCF_000308455.1_ASM30845v1/GCF_000308455.1_ASM30845v1_genomic.fna.gz | <https://ftp.ncbi.nlm.nih.gov/genomes/all/GCF/000/308/455/GCF_000308455.1_ASM30845v1/GCF_000308455.1_ASM30845v1_genomic.gff.gz> | https://ftp.ncbi.nlm.nih.gov/genomes/all/GCF/000/308/455/GCF_000308455.1_ASM30845v1/GCF_000308455.1_ASM30845v1_cds_from_genomic.fna.gz | Generic sample from *Nocardia abscessus* NBRC 100374 | National Institute of Technology and Evaluation Biological Resource Center | https://www.ncbi.nlm.nih.gov/data-hub/assembly/GCF_000308455.1/ |  |
| *Nocardia_araoensi-s* | 228600 | araoensis | https://ftp.ncbi.nlm.nih.gov/genomes/all/GCF/000/308/435/GCF_000308435.1_ASM30843v1/GCF_000308435.1_ASM30843v1_protein.faa.gz | https://ftp.ncbi.nlm.nih.gov/genomes/all/GCF/000/308/435/GCF_000308435.1_ASM30843v1/GCF_000308435.1_ASM30843v1_genomic.fna.gz | https://ftp.ncbi.nlm.nih.gov/genomes/all/GCF/000/308/435/GCF_000308435.1_ASM30843v1/GCF_000308435.1_ASM30843v1_genomic.gff.gz | https://ftp.ncbi.nlm.nih.gov/genomes/all/GCF/000/308/435/GCF_000308435.1_ASM30843v1/GCF_000308435.1_ASM30843v1_cds_from_genomic.fna.gz | Generic sample from *Nocardia araoensis* NBRC 100135 | National Institute of Technology and Evaluation Biological Resource Center | https://www.ncbi.nlm.nih.gov/data-hub/assembly/GCF_000308435.1/ |  |
| *Nocardia_asiatica* | 209252 | asiatica | https://ftp.ncbi.nlm.nih.gov/genomes/all/GCF/000/308/415/GCF_000308415.1_ASM30841v1/GCF_000308415.1_ASM30841v1_protein.faa.gz | https://ftp.ncbi.nlm.nih.gov/genomes/all/GCF/000/308/415/GCF_000308415.1_ASM30841v1/GCF_000308415.1_ASM30841v1_genomic.fna.gz | https://ftp.ncbi.nlm.nih.gov/genomes/all/GCF/000/308/415/GCF_000308415.1_ASM30841v1/GCF_000308415.1_ASM30841v1_genomic.gff.gz | https://ftp.ncbi.nlm.nih.gov/genomes/all/GCF/000/308/415/GCF_000308415.1_ASM30841v1/GCF_000308415.1_ASM30841v1_cds_from_genomic.fna.gz | Generic sample from *Nocardia asiatica* NBRC 100129 | National Institute of Technology and Evaluation Biological Resource Center | https://www.ncbi.nlm.nih.gov/data-hub/assembly/GCF_000308415.1/ |  |
| *Nocardia_aobensis* | 257277 | aobensis | https://ftp.ncbi.nlm.nih.gov/genomes/all/GCF/000/308/375/GCF_000308375.1_ASM30837v1/GCF_000308375.1_ASM30837v1_protein.faa.gz | https://ftp.ncbi.nlm.nih.gov/genomes/all/GCF/000/308/375/GCF_000308375.1_ASM30837v1/GCF_000308375.1_ASM30837v1_genomic.fna.gz | https://ftp.ncbi.nlm.nih.gov/genomes/all/GCF/000/308/375/GCF_000308375.1_ASM30837v1/GCF_000308375.1_ASM30837v1_genomic.gff.gz | https://ftp.ncbi.nlm.nih.gov/genomes/all/GCF/000/308/375/GCF_000308375.1_ASM30837v1/GCF_000308375.1_ASM30837v1_cds_from_genomic.fna.gz | Generic sample from *Nocardia aobensis* NBRC 100429 | National Institute of Technology and Evaluation Biological Resource Center | https://www.ncbi.nlm.nih.gov/data-hub/assembly/GCF_000308375.1/ |  |
| *Nocardia_asteroide-s* | 1824 | *Nocardia_aste-roides* | <https://ftp.ncbi.nlm.nih.gov/genomes/all/GCF/000/308/355/GCF_000308355.2_ASM30835v2/GCF_000308355.2_ASM30835v2_protein.faa.gz> | https://ftp.ncbi.nlm.nih.gov/genomes/all/GCF/000/308/355/GCF_000308355.2_ASM30835v2/GCF_000308355.2_ASM30835v2_genomic.fna.gz | https://ftp.ncbi.nlm.nih.gov/genomes/all/GCF/000/308/355/GCF_000308355.2_ASM30835v2/GCF_000308355.2_ASM30835v2_genomic.gff.gz | https://ftp.ncbi.nlm.nih.gov/genomes/all/GCF/000/308/355/GCF_000308355.2_ASM30835v2/GCF_000308355.2_ASM30835v2_cds_from_genomic.fna.gz | Generic sample from *Nocardia asteroides* NBRC 15531 | National Institute of Technology and Evaluation Biological Resource Center | https://www.ncbi.nlm.nih.gov/data-hub/assembly/GCF_000308355.2/ |  |
| *Nocardia_arizonen-sis* | 1141647 | arizonensis | https://ftp.ncbi.nlm.nih.gov/genomes/all/GCF/001/310/275/GCF_001310275.1_ASM131027v1/GCF_001310275.1_ASM131027v1_protein.faa.gz | https://ftp.ncbi.nlm.nih.gov/genomes/all/GCF/001/310/275/GCF_001310275.1_ASM131027v1/GCF_001310275.1_ASM131027v1_genomic.fna.gz | https://ftp.ncbi.nlm.nih.gov/genomes/all/GCF/001/310/275/GCF_001310275.1_ASM131027v1/GCF_001310275.1_ASM131027v1_genomic.gff.gz | https://ftp.ncbi.nlm.nih.gov/genomes/all/GCF/001/310/275/GCF_001310275.1_ASM131027v1/GCF_001310275.1_ASM131027v1_cds_from_genomic.fna.gz | *Nocardia arizonensis* NBRC 108935 | National Institute of Technology and Evaluation Biological Resource Center | https://www.ncbi.nlm.nih.gov/data-hub/assembly/GCF_001618405.1/ |  |
| *Nocardia_vulneris* | 1141657 | *Nocardia_vuln-eris* | https://ftp.ncbi.nlm.nih.gov/genomes/all/GCF/000/811/985/GCF_000811985.1_ASM81198v1/GCF_000811985.1_ASM81198v1_protein.faa.gz | https://ftp.ncbi.nlm.nih.gov/genomes/all/GCF/000/811/985/GCF_000811985.1_ASM81198v1/GCF_000811985.1_ASM81198v1_genomic.fna.gz | https://ftp.ncbi.nlm.nih.gov/genomes/all/GCF/000/811/985/GCF_000811985.1_ASM81198v1/GCF_000811985.1_ASM81198v1_genomic.gff.gz | https://ftp.ncbi.nlm.nih.gov/genomes/all/GCF/000/811/985/GCF_000811985.1_ASM81198v1/GCF_000811985.1_ASM81198v1_cds_from_genomic.fna.gz | Type strain of *Nocardia vulneris* | National Institute of Technology and Evaluation Biological Resource Center | https://www.ncbi.nlm.nih.gov/data-hub/assembly/GCF_001613425.1/ |  |
| *Nocardia_fluminea* | 134984 | *Nocardia_flum-inea* | https://ftp.ncbi.nlm.nih.gov/genomes/all/GCF/002/846/365/GCF_002846365.1_ASM284636v1/GCF_002846365.1_ASM284636v1_protein.faa.gz | https://ftp.ncbi.nlm.nih.gov/genomes/all/GCF/002/846/365/GCF_002846365.1_ASM284636v1/GCF_002846365.1_ASM284636v1_genomic.fna.gz | https://ftp.ncbi.nlm.nih.gov/genomes/all/GCF/002/846/365/GCF_002846365.1_ASM284636v1/GCF_002846365.1_ASM284636v1_genomic.gff.gz | https://ftp.ncbi.nlm.nih.gov/genomes/all/GCF/002/846/365/GCF_002846365.1_ASM284636v1/GCF_002846365.1_ASM284636v1_cds_from_genomic.fna.gz | *Nocardia fluminea* DSM 44489 | DOE Joint Genome Institute | https://www.ncbi.nlm.nih.gov/data-hub/assembly/GCF_002846365.1/ |  |
| *Nocardia_panacis* | 2340916 | panacis | https://ftp.ncbi.nlm.nih.gov/genomes/all/GCF/003/598/715/GCF_003598715.1_ASM359871v1/GCF_003598715.1_ASM359871v1_protein.faa.gz | https://ftp.ncbi.nlm.nih.gov/genomes/all/GCF/003/598/715/GCF_003598715.1_ASM359871v1/GCF_003598715.1_ASM359871v1_genomic.fna.gz | https://ftp.ncbi.nlm.nih.gov/genomes/all/GCF/003/598/715/GCF_003598715.1_ASM359871v1/GCF_003598715.1_ASM359871v1_genomic.gff.gz | https://ftp.ncbi.nlm.nih.gov/genomes/all/GCF/003/598/715/GCF_003598715.1_ASM359871v1/GCF_003598715.1_ASM359871v1_cds_from_genomic.fna.gz | Microbe sample from *Nocardia panacis* | Yunnan Institute of Microbiology | https://www.ncbi.nlm.nih.gov/data-hub/assembly/GCF_003598715.1/ |  |
| *Nocardia_stercoris* | 2483361 | stercoris | https://ftp.ncbi.nlm.nih.gov/genomes/all/GCF/003/696/265/GCF_003696265.1_ASM369626v1/GCF_003696265.1_ASM369626v1_protein.faa.gz | https://ftp.ncbi.nlm.nih.gov/genomes/all/GCF/003/696/265/GCF_003696265.1_ASM369626v1/GCF_003696265.1_ASM369626v1_genomic.fna.gz | https://ftp.ncbi.nlm.nih.gov/genomes/all/GCF/003/696/265/GCF_003696265.1_ASM369626v1/GCF_003696265.1_ASM369626v1_genomic.gff.gz | https://ftp.ncbi.nlm.nih.gov/genomes/all/GCF/003/696/265/GCF_003696265.1_ASM369626v1/GCF_003696265.1_ASM369626v1_cds_from_genomic.fna.gz | Microbe sample from *Nocardia stercoris* | School of life science | https://www.ncbi.nlm.nih.gov/data-hub/assembly/GCF_003696265.1/ |  |
| *Nocardia_mangyae-nsis* | 2213200 | *Nocardia_man-gyaensis* | https://ftp.ncbi.nlm.nih.gov/genomes/all/GCF/001/886/715/GCF_001886715.1_ASM188671v1/GCF_001886715.1_ASM188671v1_protein.faa.gz | https://ftp.ncbi.nlm.nih.gov/genomes/all/GCF/001/886/715/GCF_001886715.1_ASM188671v1/GCF_001886715.1_ASM188671v1_genomic.fna.gz | https://ftp.ncbi.nlm.nih.gov/genomes/all/GCF/001/886/715/GCF_001886715.1_ASM188671v1/GCF_001886715.1_ASM188671v1_genomic.gff.gz | https://ftp.ncbi.nlm.nih.gov/genomes/all/GCF/001/886/715/GCF_001886715.1_ASM188671v1/GCF_001886715.1_ASM188671v1_cds_from_genomic.fna.gz | Microbe sample from *Nocardia mangyaensis* | Northwest Institute of Eco-Environment and Resources,CAS | https://www.ncbi.nlm.nih.gov/data-hub/assembly/GCF_001886715.1/ |  |
| *Nocardia_bhagyanarayanae* | 1215925 | bhagyanarayanae | https://ftp.ncbi.nlm.nih.gov/genomes/all/GCF/006/716/565/GCF_006716565.1_ASM671656v1/GCF_006716565.1_ASM671656v1_protein.faa.gz | https://ftp.ncbi.nlm.nih.gov/genomes/all/GCF/006/716/565/GCF_006716565.1_ASM671656v1/GCF_006716565.1_ASM671656v1_genomic.fna.gz | https://ftp.ncbi.nlm.nih.gov/genomes/all/GCF/006/716/565/GCF_006716565.1_ASM671656v1/GCF_006716565.1_ASM671656v1_genomic.gff.gz | https://ftp.ncbi.nlm.nih.gov/genomes/all/GCF/006/716/565/GCF_006716565.1_ASM671656v1/GCF_006716565.1_ASM671656v1_cds_from_genomic.fna.gz | *Nocardia bhagyanarayan-ae* DSM 103495 | DOE Joint Genome Institute | https://www.ncbi.nlm.nih.gov/data-hub/assembly/GCF_006716565.1/ |  |
| *Nocardia_suismass-iliense* | 2077092 | suismassiliense | https://ftp.ncbi.nlm.nih.gov/genomes/all/GCF/900/269/665/GCF_900269665.1_PRJEB24498/GCF_900269665.1_PRJEB24498_protein.faa.gz | https://ftp.ncbi.nlm.nih.gov/genomes/all/GCF/900/269/665/GCF_900269665.1_PRJEB24498/GCF_900269665.1_PRJEB24498_genomic.fna.gz | https://ftp.ncbi.nlm.nih.gov/genomes/all/GCF/900/269/665/GCF_900269665.1_PRJEB24498/GCF_900269665.1_PRJEB24498_genomic.gff.gz | https://ftp.ncbi.nlm.nih.gov/genomes/all/GCF/900/269/665/GCF_900269665.1_PRJEB24498/GCF_900269665.1_PRJEB24498_cds_from_genomic.fna.gz | *Nocardia suismassiliense* genome | EBI | https://www.ncbi.nlm.nih.gov/data-hub/assembly/GCF_900269665.1/ |  |
| *Nocardia_donostie-nsis* | 1538463 | donostiensis | https://ftp.ncbi.nlm.nih.gov/genomes/all/GCF/002/081/795/GCF_002081795.1_ASM208179v1/GCF_002081795.1_ASM208179v1_protein.faa.gz | https://ftp.ncbi.nlm.nih.gov/genomes/all/GCF/002/081/795/GCF_002081795.1_ASM208179v1/GCF_002081795.1_ASM208179v1_genomic.fna.gz | https://ftp.ncbi.nlm.nih.gov/genomes/all/GCF/002/081/795/GCF_002081795.1_ASM208179v1/GCF_002081795.1_ASM208179v1_genomic.gff.gz | https://ftp.ncbi.nlm.nih.gov/genomes/all/GCF/002/081/795/GCF_002081795.1_ASM208179v1/GCF_002081795.1_ASM208179v1_cds_from_genomic.fna.gz | Pathogen: clinical or host-associated sample from *Nocardia donostiensis* | CDC | https://www.ncbi.nlm.nih.gov/data-hub/assembly/GCF_002081795.1/ |  |
| *Nocardia_crassostr-eae* | 53428 | crassostreae | https://ftp.ncbi.nlm.nih.gov/genomes/all/GCF/001/613/405/GCF_001613405.1_ASM161340v1/GCF_001613405.1_ASM161340v1_protein.faa.gz | https://ftp.ncbi.nlm.nih.gov/genomes/all/GCF/001/613/405/GCF_001613405.1_ASM161340v1/GCF_001613405.1_ASM161340v1_genomic.fna.gz | https://ftp.ncbi.nlm.nih.gov/genomes/all/GCF/001/613/405/GCF_001613405.1_ASM161340v1/GCF_001613405.1_ASM161340v1_genomic.gff.gz | https://ftp.ncbi.nlm.nih.gov/genomes/all/GCF/001/613/405/GCF_001613405.1_ASM161340v1/GCF_001613405.1_ASM161340v1_cds_from_genomic.fna.gz | Type strain of *Nocardia crassostreae* | National Institute of Technology and Evaluation Biological Resource Center | https://www.ncbi.nlm.nih.gov/data-hub/assembly/GCF_001613405.1/ |  |
| *Nocardia_salmonic-ida* | 53431 | *Nocardia_sal-monicida* | <https://ftp.ncbi.nlm.nih.gov/genomes/all/GCF/001/613/085/GCF_001613085.1_ASM161308v1/GCF_001613085.1_ASM161308v1_protein.faa.gz> | https://ftp.ncbi.nlm.nih.gov/genomes/all/GCF/001/613/085/GCF_001613085.1_ASM161308v1/GCF_001613085.1_ASM161308v1_genomic.fna.gz | <https://ftp.ncbi.nlm.nih.gov/genomes/all/GCF/001/613/085/GCF_001613085.1_ASM161308v1/GCF_001613085.1_ASM161308v1_genomic.gff.gz> | https://ftp.ncbi.nlm.nih.gov/genomes/all/GCF/001/613/085/GCF_001613085.1_ASM161308v1/GCF_001613085.1_ASM161308v1_cds_from_genomic.fna.gz | *Nocardia salmonicida* NBRC 13393 | National Institute of Technology and Evaluation Biological Resource Center | https://www.ncbi.nlm.nih.gov/data-hub/assembly/GCF_001613085.1/ |  |
| *Nocardia_brasilien-sis* | 37326 | *Nocardia_bras-iliensis* | https://ftp.ncbi.nlm.nih.gov/genomes/all/GCF/000/250/675/GCF_000250675.2_ASM25067v3/GCF_000250675.2_ASM25067v3_protein.faa.gz | <https://ftp.ncbi.nlm.nih.gov/genomes/all/GCF/000/250/675/GCF_000250675.2_ASM25067v3/GCF_000250675.2_ASM25067v3_genomic.fna.gz> | https://ftp.ncbi.nlm.nih.gov/genomes/all/GCF/000/250/675/GCF_000250675.2_ASM25067v3/GCF_000250675.2_ASM25067v3_genomic.gff.gz | https://ftp.ncbi.nlm.nih.gov/genomes/all/GCF/000/250/675/GCF_000250675.2_ASM25067v3/GCF_000250675.2_ASM25067v3_cds_from_genomic.fna.gz | Pathogen: clinical or host-associated sample from *Nocardia brasiliensis* | US Food and Drug Administration | https://www.ncbi.nlm.nih.gov/data-hub/assembly/GCF_002209125.2/ |  |
| *Nocardia_farcinica* | 37329 | *Nocardia_farc-inica* | <https://ftp.ncbi.nlm.nih.gov/genomes/all/GCF/001/182/745/GCF_001182745.1_NCTC11134/GCF_001182745.1_NCTC11134_protein.faa.gz> | https://ftp.ncbi.nlm.nih.gov/genomes/all/GCF/001/182/745/GCF_001182745.1_NCTC11134/GCF_001182745.1_NCTC11134_genomic.fna.gz | https://ftp.ncbi.nlm.nih.gov/genomes/all/GCF/001/182/745/GCF_001182745.1_NCTC11134/GCF_001182745.1_NCTC11134_genomic.gff.gz | https://ftp.ncbi.nlm.nih.gov/genomes/all/GCF/001/182/745/GCF_001182745.1_NCTC11134/GCF_001182745.1_NCTC11134_cds_from_genomic.fna.gz | NCTC11134 | EBI | https://www.ncbi.nlm.nih.gov/data-hub/assembly/GCF_001182745.1/ |  |
| *Nocardia_nova* | 37330 | *Nocardia_nov-a* | <ftp://ftp.ncbi.nlm.nih.gov/genomes/all/GCF/000/523/235/GCF_000523235.1_ASM52323v1/GCF_000523235.1_ASM52323v1_protein.faa.gz> | ftp://ftp.ncbi.nlm.nih.gov/genomes/all/GCF/000/523/235/GCF_000523235.1_ASM52323v1/GCF_000523235.1_ASM52323v1_genomic.fna.gz | ftp://ftp.ncbi.nlm.nih.gov/genomes/all/GCF/000/523/235/GCF_000523235.1_ASM52323v1/GCF_000523235.1_ASM52323v1_genomic.gff.gz | ftp://ftp.ncbi.nlm.nih.gov/genomes/all/GCF/000/523/235/GCF_000523235.1_ASM52323v1/GCF_000523235.1_ASM52323v1_cds_from_genomic.fna.gz | *Nocardia nova* LGO-A14 | University of Cambridge | https://www.ncbi.nlm.nih.gov/data-hub/assembly/GCF_019219615.1/ |  |
| *Nocardia_otitidisca-viarum* | 1823 | *Nocardia_otiti-discaviarum* | <https://ftp.ncbi.nlm.nih.gov/genomes/all/GCF/000/308/635/GCF_000308635.1_ASM30863v1/GCF_000308635.1_ASM30863v1_protein.faa.gz> | https://ftp.ncbi.nlm.nih.gov/genomes/all/GCF/000/308/635/GCF_000308635.1_ASM30863v1/GCF_000308635.1_ASM30863v1_genomic.fna.gz | https://ftp.ncbi.nlm.nih.gov/genomes/all/GCF/000/308/635/GCF_000308635.1_ASM30863v1/GCF_000308635.1_ASM30863v1_genomic.gff.gz | https://ftp.ncbi.nlm.nih.gov/genomes/all/GCF/000/308/635/GCF_000308635.1_ASM30863v1/GCF_000308635.1_ASM30863v1_cds_from_genomic.fna.gz | Microbe sample from *Nocardia otitidiscaviarum* | NEB | https://www.ncbi.nlm.nih.gov/data-hub/assembly/GCF_007362295.1/ |  |
